# Thermochemical Performance Analysis of the Steam Reforming of Methane in a Fixed Bed Membrane Reformer: A Modelling and Simulation Study

**DOI:** 10.3390/membranes11010006

**Published:** 2020-12-23

**Authors:** João Paulo Fernando de Medeiros, Vitória da Fonseca Dias, José Marcelo da Silva, Jornandes Dias da Silva

**Affiliations:** Laboratory of Environmental and Energetic Technology, Polytechnic School—UPE, Rua Benfica-455, Madalena, Recife, PE 50750-470, Brazil; joaopaulofernandodemedeiros@gmail.com (J.P.F.d.M.); vi.dias11@hotmail.com (V.d.F.D.); josemarcelo@eletronenergy.com.br (J.M.d.S.)

**Keywords:** membrane reformer, physical-mathematical model, steam reforming, Pd-based membrane, hydrogen production

## Abstract

Pd-based membrane reformers have been substantially studied in the past as a promising reformer to produce high-purity H_2_ from thermochemical conversion of methane (CH_4_). A variety of research approaches have been taken in the experimental and theoretical fields. The main objective of this work is a theoretical modelling to describe the process variables of the Steam Reforming of Methane (SRM) method on the Pd-based membrane reformer. These process variables describe the specific aims of each equation of the mathematical model characterizing the performance from reformer. The simulated results of the mole fractions of components (MFCs) at the outlet of the Fixed Bed Reformer (FBR) and Packed-Bed Membrane Reformer (PBMR) have been validated. When the H_2_O/CH_4_ ratio decreases in PBMR, the Endothermic Reaction Temperature (ERT) is notably increased (998.32 K) at the outlet of the PBMR’s reaction zone. On the other hand, when the H_2_O/CH_4_ ratio increases in PBMR, the ERT is remarkably decreased (827.83 K) at the outlet of the PBMR’s reaction zone. An increase of the spatial velocity (S_sp_) indicates a reduction in the residence time of reactant molecules inside PBMR and, thus, a decrease of the ERT and conversion of CH_4_. In contrast, a reduction of the S_sp_ shows an increase of the residence time of reactant molecules within PBMR and, therefore, a rise of the ERT and conversion of CH_4_. An increase of the H_2_O/CH_4_ ratio raises the conversion rate (CR) of CH_4_ due to the reduction of the coke content on the catalyst particles. Conversely, a reduction of the H_2_O/CH_4_ ratio decreases the CR of CH_4_ owing to the increase of the coke content on the catalyst particles. Contrary to the CR of CH_4_, the consumption-based yield (CBY) of H_2_ sharply decreases with the increase of the H_2_O/CH_4_ ratio. An increase of the ERT raises the thermochemical energy storage efficiency (η_tese_) from 68.96% (ERT = 1023 K), 63.21% (ERT = 973 K), and 48.12% (ERT = 723 K). The chemical energy, sensible heat, and heat loss reached values of 384.96 W, 151.68 W, and 249.73 W at 973 K. The selectivity of H_2_ presents higher amounts in the gaseous mixture that varies from 60.98 to 73.18 while CH_4_ showed lower values ranging from 1.41 to 2.06. Our work is limited to the SRM method. In terms of future uses of this method, new works can be undertaken using novel materials (open-cell foams) and the physical-mathematical model (two-dimensional and three-dimensional) to evaluate the concentration polarization inside membrane reactors.

## 1. Introduction

The production of hydrogen (H_2_) can be carried out through different methods such as thermochemical method (heat and chemical reactions to produce H_2_), reforming of hydrocarbons, biomass gasification, coal gasification, electrolytic method, and biological method. Usually, the thermochemical reforming methods are used to study the Thermochemical Energy Storage (TES) technology of H_2_. The TES of H_2_ can be produced from reforming reactions with high energy involved in chemical reaction. In particular, H_2_ can be obtained from thermochemical conversion of CH_4_ by driving endothermic reaction methods as the SRM and/or dry reforming of methane (DRM) [1]. The choice of the SRM or DRM depends of the desired ending product. If H_2_ is the desired ending product, then the SRM is preferable. H_2_ can also provide environmental benefits as a promising renewable energy vector which can be used in several industrial processes [2,3]. Technological development efforts to produce renewable energy have been reported since last century. H_2_ is one of the most important energy vectors in the universe accounting for almost 75% of the all universe mass. H_2_ is considered a clean energy source for the future.

Membrane reformers (MRs) can be used to produce H_2_ and purify it in packed-bed and/or fluidized-bed setups [4,5]. The production of H_2_ inside MRs is still attracting the interest of researchers and engineers. This article is focused on the thermochemical conversion of CH_4_ in a Packed-Bed Membrane Reformer (PBMR). In this setup, H_2_ gas from shell side (reaction system) passes towards a permeation side through a Pd-based dense membrane [6]. The permselectivity properties of a Pd-based dense membrane such as permeability and selectivity are able to enhance the production of H_2_ from the thermochemical reforming reactions. The Pd-based dense membrane can act as a remover and it facilitates the selective removal of the gaseous molecule of H_2_. The removal of H_2_ through Pd-based dense membrane shifts the equilibrium of reforming reactions to the chosen direction according to the Le Chatelier principle [7,8,9]. PBMRs offer many potential advantages such as enhanced conversion of hydrocarbons, reduced cost, improved yield, and high selectivity.

PBMR is an apparatus which integrates the reaction zone and permeation zone separated by a Pd-based dense membrane on the same physical equipment. The Pd-based dense membrane is a barrier that allows only certain component (H_2_) to pass through it and it acts as separator [10,11]. Among the metals, Pd and its alloys have been applied for manufacturing H_2_ separators as a function of their high membrane permeability towards H_2_. The mathematical modelling from PBMRs is important to design and optimize this type reformer in order to understand its behavior for a given reaction system. The mathematical models of PBMRs (gas-solid system) can be developed using mass, energy and momentum balance equations for the gas phase as well as mass, energy and momentum balance equations to the solid phase [3,6,12,13,14]. The dynamic performance of a state variable inside PBMRs can be investigated from an initial value up to a steady state of this variable. In this work, a Non-isothermal Pseudo-Homogeneous Dynamic (NIPHD) model is used to model the SRM method inside PBMR. The NIPHD model is described by a system of Nonlinear Partial Differential Equations (NPDEs) that couple to a complex kinetic model of the SRM.

NIPHD models can be used to simulate and analyze the SRM method inside PBMR. The application of NIPHD models can be an excellent alternative to predict faster solutions of systems of NPDEs due to the lower computational time [15,16]. In this context, the NIPHD model is able to predict the main characteristics of the SRM method’s dynamic performance in PBMR [17]. In addition, the system of NPDEs represents a strong tool for facilitating the project, optimization, and PBMR reformer’s control [3,18,19,20]. The numerical solution of the system of NPDEs has been a great challenge due to the numerical stability. Given this, several numerical methodologies have been used to compute numerical solutions [21,22]. The choice of methodology is dependent on the desired accuracy of the stability and robustness of numerical results of the NPDE system to maintain computational efficiency. The NPDE system of this work has been transformed into a simpler Nonlinear Ordinary Differential Equations (NODEs) system using the Coupled Integral Equation Approach (CIEA) [23,24]. The NODE system was solved by the Runge-Kutta Gill method as well as the NODE from the permeation zone.

With the purpose of reducing the research cost and project time, mathematical modelling and computer simulation are extensively used to obtain a better understanding of design parameters in reformers. The approach and solution of physical-mathematical models are still a novelty of membrane reformers to obtain sustainable clean H_2_ and, thus, the topic is a very relevant in the literature. In comparison with traditional methodologies such as finite element, finite volume, etc., that have already been used before in the literature, our methodology can provide results faster than traditional methods and, therefore, the novelty of the present work lies in the determination of the solution method. A comparative analysis had been driven to investigate ERT, CR of CH_4_, and feed-based yield (FBY) of H_2_ inside FBR and PBMR. The effects of the H_2_O/CH_4_ ratio and S_sp_ on the ERT were numerically investigated in PBMR. After checking the effects of the H_2_O/CH_4_ ratio and S_sp_ on the ERT, the effects of these parameters were also studied on the CR of CH_4_ and CBY of H_2_. In addition, the ERT’s effect was verified on the η_tese_, chemical energy, sensible heat, and heat loss. In addition, the selectivity of components (H_2_, CO, CO_2_, and CH_4_) was computed in PBMR.

## 2. Physical-Mathematical Model

**Figure 1:**
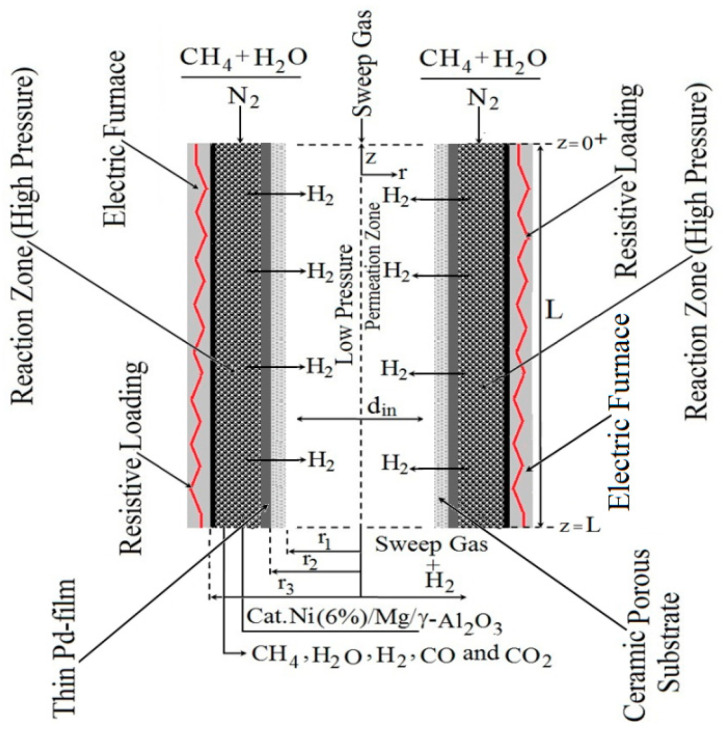
Schematic setup of the physical setup from Packed-Bed Membrane Reformer (PBMR) to study the Steam Reforming of Methane (SRM) method using electric heating.

A schematic setup is used to study the SRM method’s thermochemical conversion in PBMR according to Figure 1. The simplified setup from Figure 1 involves a heating module (electric furnace), input reagents (CH_4_, H_2_O), Sweep gas (N_2_), reaction zone, permeation zone, and outlet products (CH_4_, H_2_O, H_2_, CO, and CO_2_). The physical setup of the PBMR is built by two concentric tubes according to Figure 1. The inner tube consists of a thin palladium (Pd) dense membrane which contains a permeation zone receiving H_2_ from the reaction zone through the Pd-based dense membrane. The catalyst loading is placed between the tubes in the annular zone, named the fixed-bed.

### 2.1. Electric Power of the Electric Furnace

In Figure 1, a resistive loading inside of an electric furnace has been used to heat the FBMR’s reaction zone and therefore the thermal energy storage is used to drive the reforming reactions. The electric power provided by the electric furnace is given for Equation (1) as follows.

(1) $$P_{\mathrm{elet}.}=R\;i_{\mathrm{elet}.}^{2}=U_{\mathrm{ghe}}S_{\mathrm{she}}\left(T_{\mathrm{er}}-T_{g}\right)$$

The thermochemical energy storage is obtained by subtracting the product enthalpy (reaction heat) from the reagent enthalpy at room temperature. Thus, the PBMR’s chemical energy is obtained using Equation (2) as follows.

(2) $$Q_{\mathrm{che}}=\rho_{s}\left(\frac{1-\varepsilon_{p}}{\varepsilon_{p}}\right)\sum_{j=1}^{3}\left(\pm \Delta H_{j}\right)\eta_{j}R_{j}$$

After cooling the products to the room temperature, the sensible heat of the products (ranging from outer temperature to the room temperature) can be used and therefore the sensible heat can be computed from Equation (3) as follows.

(3) $$Q_{\mathrm{sh}}=\sum_{i=1}^{2}\int_{298K}^{T_{\mathrm{op}}}\rho_{g,i}C_{p,g,i}F_{i}\mathrm{dT};\;i=H_{2}\;\mathrm{and}\;\mathrm{CO}$$

From Equations (1)–(3), it is possible to estimate the heat loss using Equation (4) as follows.

(4) $$Q_{\mathrm{Loss}}=P_{\mathrm{elet}.}-Q_{\mathrm{che}}-Q_{\mathrm{sh}}$$

### 2.2. Thermochemical Kinetic Model

The reforming reactions of CH_4_ are used to produce syngas (H_2_ and CO) and they are highly endothermic [3]. The SRM method has a limited equilibrium and it comprises three major reactions as follows.

(5) $$\mathrm{SRM}:{\mathrm{CH}}_{4\left(g\right)}+H_{2}O_{\left(g\right)}↔{\mathrm{CO}}_{\left(g\right)}+3H_{2\left(g\right)};\;\Delta H_{298K}^{0}=205.8\;\mathrm{kJ}/\mathrm{mol}$$

(6) $$\mathrm{WGSR}:{\mathrm{CO}}_{\left(g\right)}+H_{2}O_{\left(g\right)}↔{\mathrm{CO}}_{2\left(g\right)}+H_{2\left(g\right)};\;\Delta H_{298K}^{0}=-41.1\;\mathrm{kJ}/\mathrm{mol}$$

(7) $$\mathrm{Global}\;\mathrm{SRM}:{\mathrm{CH}}_{4\left(g\right)}+2H_{2}O_{\left(g\right)}↔{\mathrm{CO}}_{2\left(g\right)}+4H_{2\left(g\right)};\;\Delta H_{298K}^{0}=164.9\;\mathrm{kJ}/\mathrm{mol}$$

The two reforming reactions, Equations (5) and (7), are highly endothermic reactions and they need high temperatures to obtain a high H_2_ productivity. On the other hand, Equation (6) is a slightly exothermic reaction and it works at low temperature when comparing to Equations (5) and (7).

The global rate equations of the three reactions, Equations (5)–(7), are based on the Langmuir-Hinshelwood kinetic model [3]. The kinetic rates from Equations (5)–(7) are considered more general for nickel (Ni) catalyst and, therefore, the equations of the SRM method are presented as:

(8) $$R_{1}=\frac{\frac{k_{1}}{P_{H_{2}}^{2.5}}\left(P_{{\mathrm{CH}}_{4}}P_{H_{2}O}-\frac{P_{H_{2}}^{3}P_{\mathrm{CO}}}{K_{\mathrm{eq}.,1}}\right)}{{\left(\beta \right)}^{2}}$$

(9) $$R_{2}=\frac{\frac{k_{2}}{P_{H_{2}}}\left(P_{\mathrm{CO}}P_{H_{2}O}-\frac{P_{H_{2}}P_{{\mathrm{CO}}_{2}}}{K_{\mathrm{eq}.,2}}\right)}{{\left(\beta \right)}^{2}}$$

(10) $$R_{3}=\frac{\frac{k_{3}}{P_{H_{2}}^{3.5}}\left(P_{{\mathrm{CH}}_{4}}P_{H_{2}O}-\frac{P_{H_{2}}^{4}P_{{\mathrm{CO}}_{2}}}{K_{\mathrm{eq}.,3}}\right)}{{\left(\beta \right)}^{2}}$$

where β is given by Equation (11) as follows.

(11) $$\beta =1+\frac{F_{H_{2}O,0}}{F_{{\mathrm{CH}}_{4},0}}+\frac{F_{H_{2},0}}{F_{{\mathrm{CH}}_{4},0}}+K_{\mathrm{CO}}P_{\mathrm{CO}}+K_{H_{2}}P_{H_{2}}+K_{{\mathrm{CH}}_{4}}P_{{\mathrm{CH}}_{4}}+\frac{K_{H_{2}O}P_{H_{2}O}}{P_{H_{2}}}$$

The partial pressures of chemical components i, i = CH_4_, H_2_O, CO, CO_2_ and H_2_, from Equations (8)–(11) are computed from Equations (12)–(16) below.

(12) $$P_{{\mathrm{CH}}_{4}}=\frac{1-\left(1-\frac{F_{{\mathrm{CH}}_{4}}}{F_{{\mathrm{CH}}_{4},0}}\right)}{\sigma }$$

(13) $$P_{H_{2}O}=\frac{\frac{F_{H_{2}O,0}}{F_{{\mathrm{CH}}_{4},0}}-\left(1-\frac{F_{{\mathrm{CH}}_{4}}}{F_{{\mathrm{CH}}_{4},0}}\right)-\left(\frac{F_{{\mathrm{CO}}_{2}}}{F_{{\mathrm{CH}}_{4},0}}\right)}{\sigma }$$

(14) $$P_{\mathrm{CO}}=\frac{\frac{F_{\mathrm{CO},0}}{F_{{\mathrm{CH}}_{4},0}}+\left(1-\frac{F_{{\mathrm{CH}}_{4}}}{F_{{\mathrm{CH}}_{4},0}}\right)-\left(\frac{F_{{\mathrm{CO}}_{2}}}{F_{{\mathrm{CH}}_{4},0}}\right)}{\sigma }$$

(15) $$P_{{\mathrm{CO}}_{2}}=\frac{\frac{F_{{\mathrm{CO}}_{2},0}}{F_{{\mathrm{CH}}_{4},0}}+\left(\frac{F_{{\mathrm{CO}}_{2}}}{F_{{\mathrm{CH}}_{4},0}}\right)}{\sigma }$$

(16) $$P_{H_{2}}=\frac{\frac{F_{H_{2},0}}{F_{{\mathrm{CH}}_{4},0}}+3\left(1-\frac{F_{{\mathrm{CH}}_{4}}}{F_{{\mathrm{CH}}_{4},0}}\right)-\left(\frac{F_{{\mathrm{CO}}_{2}}}{F_{{\mathrm{CH}}_{4},0}}\right)-\frac{F_{H_{2}}}{F_{{\mathrm{CH}}_{4},0}}}{\sigma }$$

where,

(17) $$\sigma =\frac{1+\sum_{j}^{4}\frac{F_{i,0}}{F_{{\mathrm{CH}}_{4},0}}}{P_{\mathrm{op}.}};\;j=H_{2}O,\;\mathrm{CO},\;{\mathrm{CO}}_{2}\;\mathrm{and}\;H_{2}$$

The net rates (r_i_) for each chemical component i (i = CH_4_, H_2_O, CO, CO_2_ and H_2_) are computed through Equation (18) as follows.

(18) $$r_{i}=\sum_{i=1}^{5}\sum_{j=1}^{3}\eta_{j}\sigma_{\mathrm{ij}}R_{j}$$

From Equation (18), the net rates of each chemical component i are obtained by Equations (19)–(23) as follows.

(19) $$r_{{\mathrm{CH}}_{4}}=-\eta_{1,\mathrm{av}.}R_{1}-\eta_{3,\mathrm{av}.}R_{3}$$

(20) $$r_{H_{2}O}=-\eta_{1,\mathrm{av}.}R_{1}-\eta_{2,\mathrm{av}.}R_{2}-\eta_{3,\mathrm{av}.}R_{3}$$

(21) $$r_{H_{2}}=3\eta_{1,\mathrm{av}.}R_{1}+\eta_{2,\mathrm{av}.}R_{2}+4\eta_{3,\mathrm{av}.}R_{3}$$

(22) $$r_{\mathrm{CO}}=\eta_{1,\mathrm{av}.}R_{1}-\eta_{2,\mathrm{av}.}R_{2}$$

(23) $$r_{{\mathrm{CO}}_{2}}=\eta_{2,\mathrm{av}.}R_{2}+\eta_{3,\mathrm{av}.}R_{3}$$

### 2.3. PBMR’s Mathematical Modelling

The mathematical modelling inside PBMR’s reaction zone is described through the NIPHD model. The development of the NIPHD model takes into account the following assumptions: (1) the NIPHD model is described under non-isothermal conditions inside the reaction zone, (2) the NIPHD model in the reaction zone is plug-flow with axial dispersion under transient condition, (3) the radial dispersion is negligible inside the reaction zone, (4) the gaseous mixture has constant density inside the reaction zone from PBMR, (5) the membrane is considered to be 100% H_2_-permselectivity, i.e., the selectivity of H_2_ is typically very high in dense metallic membranes, (6) the heat exchange between the reaction zone and permeation zone is negligible, (7) the molar flow rates in the reaction zone and permeation zone are constant, (8) the deposition effect of carbon at the surface of catalytic particles has been neglected, (9) the gas behavior in the reaction zone from PBMR was considered as an ideal gas mixture, (10) the bed porosity in the axial direction is considered constant, and (11) chemical reactions are assumed to take place at the surface of catalyst particles.

These premises are used to build the governing equations of the NIPHD model in PBMR’s reaction zone and permeation zone as follows.

#### 2.3.1. Energy Balance of the Gas Phase in Reaction Zone

The developed equation provides clear information to drive the temperature distribution of the gas phase in porous medium from PBMR’s reaction zone. The energy transport in the gas phase inside the reaction zone is characterized by a balance equation in PBMR’s axial direction. Thus, a one-dimensional dynamic equation is modelled for the temperature of the gas phase as follows.

- Energy balance in the gas phase

(24) $$\rho_{g,\mathrm{mix}.}C_{p,g,\mathrm{mix}.}\left(\frac{\partial T_{g}}{\partial t}+\frac{4q_{g}}{\pi d_{\mathrm{rz}}^{2}}\frac{\partial T_{g}}{\partial z}\right)=\lambda_{g,\mathrm{eff}}\frac{\partial^{2}T_{g}}{{\partial z}^{2}}-h_{\mathrm{gs}}\frac{\left(1-\varepsilon_{b}\right)}{\varepsilon_{b}}\frac{6}{d_{p}}\left(T_{g}-T_{\mathrm{er}}\right)$$

The gas phase’s effective thermal conductivity is defined as a function of the gaseous mixture thermal conductivity as follows.

(25) $$\lambda_{g,\mathrm{eff}.}=\varepsilon_{b}\lambda_{g,\mathrm{mix}.}$$

where

(26) $$\lambda_{g,\mathrm{mix}.}=1.52\times 10^{-11}T_{g,0}^{3}-4.86\times 10^{-8}T_{g,0}^{2}+1.02\times 10^{-4}T_{g,0}-3.93\times 10^{-3}$$

The suitable initial and boundary conditions from Equations are given as follows.

- Initial condition, i.e., t = 0

(27) $${T_{g}|}_{t=0,\;0\leq z\leq L}=0$$

- At the inlet face surface (upper) of the reaction zone from PBMR, i.e., z = 0^+^

(28) $${\frac{\partial T_{g}}{\partial z}|}_{z=0^{+},\;t\geq 0}^{\mathrm{Up}.}=\frac{\rho_{g,\mathrm{mix}.}C_{p,g,\mathrm{mix}.}}{\lambda_{g,\mathrm{mix}.}}\frac{4q_{g}}{\pi d_{\mathrm{rz}}^{2}}\left({T_{g}|}_{z=0^{+},\;t\geq 0}^{\mathrm{Up}.}-T_{g,\infty }^{\mathrm{Up}.}\right)$$

- At the outlet face surface (bottom) of the reaction zone from PBMR, i.e., z = L

(29) $${\frac{\partial T_{g}}{\partial z}|}_{z=L,\;t\geq 0}^{\mathrm{Bot}.}=\frac{h_{\mathrm{gs},\mathrm{eff}.}}{\lambda_{g,\mathrm{eff}.}}\left(T_{g,\infty }^{\mathrm{Bot}.}-{T_{g}|}_{z=L,\;t\geq 0}^{\mathrm{Bot}.}\right)$$

#### 2.3.2. Energy Balance of the Solid Phase in Reaction Zone

The spherical particle’s tortuous structure in the reaction zone could give rise to turbulences with an increase in heat transfer between the solid and gas phases. The thermal energy storage takes place on the solid particles to ensure sufficient energy for processing the endothermic reactions from the SRM method. A promising point is reported by thermal interactions at the surface of catalyst particles where SRM reactions are thermochemically converted. However, the energy balance for the temperature of reforming reactions at the surface of catalytic particles is given as follows.

- Energy balance at the surface of catalytic particles

(30) $$\rho_{s}C_{p,s}\frac{\partial T_{\mathrm{er}}}{\partial t}=\lambda_{s,\mathrm{eff}}\frac{\partial^{2}T_{\mathrm{er}}}{\partial z^{2}}+h_{\mathrm{sg}}\frac{6}{d_{p}}\frac{\left(1-\varepsilon_{b}\right)}{\varepsilon_{b}}\left(T_{g}-T_{\mathrm{er}}\right)+\rho_{s}\frac{\left(1-\varepsilon_{p}\right)}{\varepsilon_{p}}\sum_{j=1}^{3}\pm \Delta H_{j}\eta_{j}R_{j}$$

The solid phase’s effective thermal conductivity is defined as a function of the thermal conductivity of the gaseous mixture according to Equation below.

(31) $$\lambda_{s,\mathrm{eff}}=\left(1-\varepsilon_{b}\right)\lambda_{g,\mathrm{mix}.}$$

The suitable initial and boundary conditions from Equations are given as follows.

- Initial condition at t = 0

(32) $${T_{\mathrm{er}}|}_{t=0,\;0\leq z\leq L}=T_{\mathrm{er}}{}_{,0}$$

- At the inlet face surface (upper) of the reaction zone from PBMR, i.e., z = 0^+^

(33) $${\frac{\partial T_{\mathrm{er}}}{\partial z}|}_{z=0^{+},\;t\geq 0}^{\mathrm{Up}.}=\frac{q_{h}}{\lambda_{s}}$$

- At the outlet face surface (bottom) of the reaction zone from PBMR, i.e., z = L

(34) $${\frac{\partial T_{\mathrm{er}}}{\partial z}|}_{z=L,\;t\geq 0}^{\mathrm{Bot}.}=\frac{h_{\mathrm{sg},\mathrm{eff}}}{\lambda_{s,\mathrm{eff}.}}\left(T_{\mathrm{er},\infty }^{\mathrm{Bot}.}-{T_{\mathrm{er}}|}_{z=L,\;t\geq 0}^{\mathrm{Bot}.}\right)$$

#### 2.3.3. Transport Equations of Chemical Components in Reaction Zone

Based on assumptions mentioned in Section 2.3, chemical components on the reaction zone from PBMR are modelled by Equation (35). Equation (35) reports only the transport equations for chemical components i (i = CH_4_, H_2_O, CO and CO_2_) without H_2_ as follows.

- Transport equations of chemical components i in reaction zone

(35) $$\frac{u_{\mathrm{sg}}}{g}\frac{\partial F_{i}}{\partial t}+\frac{4q_{g}}{S_{\mathrm{sp}}\pi d_{\mathrm{rz}}^{2}}\frac{\partial F_{i}}{\partial z}=\frac{D_{\mathrm{ax},i}}{S_{\mathrm{sp}}}\frac{\partial^{2}F_{i}}{\partial z^{2}}+\rho_{s}r_{\mathrm{rz}}^{2}L_{z}\left(1-\varepsilon_{b}\right)r_{i};\;0<z<L$$

The suitable initial and boundary conditions from Equations are presented as follows.

- Initial condition, i.e., t = 0

(36) $${F_{i}|}_{t=0,\;0\leq z\leq L}=F_{i,0}$$

- At the inlet face surface (upper) of the reaction zone from PBMR, i.e., z = 0^+^

(37) $${\varepsilon_{b}\frac{D_{\mathrm{ax},i}}{L}\frac{\partial F_{i}}{\partial z}|}_{z=0^{+},\;t\geq 0}^{\mathrm{Up}.}=u_{\mathrm{sg}}\left({F_{i}|}_{z=0^{+},\;t\geq 0}^{\mathrm{Up}.}-F_{i,\infty }^{\mathrm{Up}.}\right)$$

- At the outlet face surface (bottom) of the reaction zone from PBMR, i.e., z = L

(38) $${\varepsilon_{b}\frac{D_{\mathrm{ax},i}}{L}\frac{\partial F_{i}}{\partial z}|}_{z=L,\;t\geq 0}^{\mathrm{Bot}.}=k_{\mathrm{gs},\mathrm{eff}}\left({F_{i}|}_{z=L,\;t\geq 0}^{\mathrm{Bot}.}-F_{i,\infty }^{\mathrm{Bot}.}\right)$$

As H_2_ permeates the Pd-based dense membrane, a transport equation is specifically developed for H_2_ inside the reaction zone. Thus, this equation is reported by Equation (39) as follows.

- Transport equation of H_2_ in reaction zone

(39) $$\frac{u_{\mathrm{sg}}}{g}\frac{\partial F_{H_{2}}}{\partial t}+\frac{4q_{g}}{S_{\mathrm{sp}}\pi d_{\mathrm{rz}}^{2}}\frac{\partial F_{H_{2}}}{\partial z}=\frac{D_{\mathrm{ax},H_{2}}}{S_{\mathrm{sp}}}\frac{\partial^{2}F_{H_{2}}}{\partial z^{2}}+\rho_{s}r_{\mathrm{rz}}^{2}L_{z}\left(1-\varepsilon_{b}\right)r_{H_{2}}-\pi d_{\mathrm{zr}}^{2}J_{H_{2},\mathrm{per}.};\;0<z<L$$

- Initial condition, i.e., t = 0

(40) $${F_{H_{2}}|}_{t=0,\;0\leq z\leq L}=F_{H_{2},0}$$

- At the inlet face surface (upper) of the reaction zone from PBMR, i.e., z = 0^+^

(41) $${\varepsilon_{b}\frac{D_{\mathrm{ax},H_{2}}}{L}\frac{\partial F_{H_{2}}}{\partial z}|}_{z=0^{+},\;t\geq 0}^{\mathrm{Up}.}=u_{\mathrm{sg}}\left({F_{H_{2}}|}_{z=0^{+},\;t\geq 0}^{\mathrm{Up}.}-F_{H_{2},\infty }^{\mathrm{Up}.}\right)$$

- At the outlet face surface (bottom) of the reaction zone from PBMR, i.e., z = L

(42) $${\varepsilon_{b}\frac{D_{\mathrm{ax},H_{2}}}{L}\frac{\partial F_{H_{2}}}{\partial z}|}_{z=L,\;t\geq 0}^{\mathrm{Bot}.}=k_{\mathrm{gs},\mathrm{eff}}\left({F_{H_{2}}|}_{z=L,\;t\geq 0}^{\mathrm{Bot}.}-F_{H_{2},\infty }^{\mathrm{Bot}.}\right)$$

#### 2.3.4. Transport of H_2_ within the Permeation Zone

The permeation rate of H_2_ through the membrane from the high-pressure zone into the permeation zone is assumed to obey the half power pressure law. However, the permeation rate of H_2_ from the reaction zone into the permeation zone is given as follows.

(43) $$J_{H_{2},\mathrm{per}.}=\frac{Q_{0}}{\delta }\exp\left(-\frac{E_{H_{2}}}{RT_{\mathrm{er}.}}\right)\left(P_{H_{2},\mathrm{rz}}^{0.5}-P_{H_{2},\mathrm{per}.}^{0.5}\right)$$

A differential model allows us to quantify the amount of H_2_ in the permeation side, but the model has to be consistent with the permeation rate which passes through the Pd-based dense membrane. Thus, a transport equation is developed to estimate the production of H_2_ in the permeation zone from PBMR as follows.

(44) $$\frac{dF_{H_{2},\mathrm{per}.}}{\mathrm{dz}}=\frac{\pi d_{\mathrm{zr}}J_{H_{2},\mathrm{per}.}}{L};\;0<z<L$$

- At the inlet face surface (upper) of the permeation zone from PBMR, i.e., z = 0^+^

(45) $${F_{H_{2},\mathrm{per}.}|}_{z=0^{+}}^{\mathrm{Up}.}=F_{H_{2},\infty }^{\mathrm{Up}.}$$

- At the outlet face surface (bottom) of the permeation zone from PBMR, i.e., z = L

(46) $${\frac{dF_{H_{2},\mathrm{per}.}}{\mathrm{dz}}|}_{z=L}^{\mathrm{Bot}.}=0$$

### 2.4. Solution of the Mathematical Model

#### 2.4.1. Application of the CIEA Method

The CIEA method can be considered as a powerful technique because of its low computer time relative to traditional methods (finite difference, finite volume, finite element, etc.). The CIEA methodology has been used to transform the NPDE system (Equations (24), (30), (35) and (39)) into an NODE system using the boundary conditions (Equations (28), (29), (33), (34), (37), (38), (41) and (42)) of each NPDE. The coefficients of Equations (47)–(50) can be found in Appendix A of this work. Thus, NODEs (Equations (47)–(50)) are reported as follows.

- Transformed NODE for the gas phase

(47) $$\begin{array}{ll} \alpha_{g,1}\frac{d{\bar{T}}_{g}\left(t\right)}{\mathrm{dt}}= & \left(\alpha_{g,2}-\lambda_{g,\mathrm{eff}.}\alpha_{g,4}\right)T_{g}\left(0,t\right)-\left(\alpha_{g,2}+\lambda_{g,\mathrm{eff}.}\alpha_{g,5}\right)T_{g}\left(L,t\right)-\alpha_{g,3}\\ & \left({\bar{T}}_{g}\left(t\right)-{\bar{T}}_{s}\left(t\right)\right)+\lambda_{g,\mathrm{eff}.}\left(\alpha_{g,4}T_{g,\infty }^{\mathrm{Up}.}+\alpha_{g,5}T_{g,\infty }^{\mathrm{Bot}.}\right) \end{array}$$

- Transformed NODE for the solid phase

(48) $$\begin{array}{ll} \frac{d{\bar{T}}_{\mathrm{er}}\left(t\right)}{\mathrm{dt}}= & \beta_{s,5}\left({\bar{T}}_{g}\left(t\right)-{\bar{T}}_{\mathrm{er}}\left(t\right)\right)+\sum_{j=1}^{3}\pm \Delta H_{j}\eta_{j}{\bar{R}}_{j}\left(t\right)-\beta_{s,1}\beta_{s,5}T_{\mathrm{er}}\left(L,t\right)+\beta_{s,1}\\ & \left(\beta_{s,5}T_{\mathrm{er},\infty }^{\mathrm{Bot}.}-\beta_{s,4}\right) \end{array}$$

- Transformed NODE for chemical components i in reaction zone

(49) $$\begin{array}{ll} \frac{d{\bar{F}}_{i}\left(t\right)}{\mathrm{dt}}= & \left(\varphi_{f,2}\varphi_{f,5}-\varphi_{f,1}\right)F_{i}\left(L,t\right)-\left(\varphi_{f,2}\varphi_{f,4}-\varphi_{f,1}\right)F_{i}\left(0,t\right)+\varphi_{f,3}{\bar{r}}_{i}\left(t\right)+\\ & \varphi_{f,2}\left(\varphi_{f,4}F_{i,\infty }^{\mathrm{Up}.}-\varphi_{f,5}F_{i,\infty }^{\mathrm{Bot}.}\right) \end{array}$$

- Transformed NODE for H_2_ in reaction zone

(50) $$\begin{array}{ll} \frac{d{\bar{F}}_{H_{2}}\left(t\right)}{\mathrm{dt}}= & \left(\vartheta_{H_{2},2}\vartheta_{H_{2},6}-\vartheta_{H_{2},1}\right)F_{H_{2}}\left(L,t\right)-\left(\vartheta_{H_{2},2}\vartheta_{H_{2},5}-\vartheta_{H_{2},1}\right)F_{H_{2}}\left(0,t\right)+\\ & \vartheta_{H_{2},3}{\bar{r}}_{H_{2}}\left(t\right)+\vartheta_{H_{2},2}\left(\vartheta_{H_{2},5}F_{H_{2},\infty }^{\mathrm{Up}.}-\vartheta_{H_{2},6}F_{H_{2},\infty }^{\mathrm{Bot}.}\right)-\vartheta_{H_{2},4} \end{array}$$

#### 2.4.2. Approximation of the Full Solution

Several numerical methods have been proposed to solve NPDE systems [15]. The numeric methodology’s selection is limited to the desired accuracy on the consistency and robustness of numerical data of the NPDE system. Regarding NODEs, Equations (47)–(50) have been solved by the Runge-Kutta Gill method as well as the NODE in the permeation zone (Equation (44)). On the other hand, the full solution is obtained from Equations (51)–(54) as follows.

- Gas phase’s full solution

(51) $$T_{g}\left(z,t\right)=\frac{1}{2}{T_{g}\left(z,t\right)|}_{t=0}+\sum_{k=1}^{\infty }{\bar{T}}_{g}\left(t_{k}\right)\sin\left(\frac{k\pi z}{L}\right)$$

- Solid phase’s full solution

(52) $$T_{\mathrm{er}}\left(z,t\right)=\frac{1}{2}{T_{\mathrm{er}}\left(z,t\right)|}_{t=0}+\sum_{k=1}^{\infty }{\bar{T}}_{\mathrm{er}}\left(t_{k}\right)\sin\left(\frac{k\pi z}{L}\right)$$

- Full solution for chemical components i

(53) $$F_{i}\left(z,t\right)=\frac{1}{2}{F_{i}\left(z,t\right)|}_{t=0}+\sum_{k=1}^{\infty }{\bar{F}}_{i}\left(t_{k}\right)\sin\left(\frac{k\pi z}{L}\right)$$

- Full solution for chemical components H_2_.

(54) $$F_{H_{2}}\left(z,t\right)=\frac{1}{2}{F_{H_{2}}\left(z,t\right)|}_{t=0}+\sum_{k=1}^{\infty }{\bar{F}}_{H_{2}}\left(t_{k}\right)\sin\left(\frac{k\pi z}{L}\right)$$

## 3. Results

### 3.1. Model Parameters for Simulations

A physical-mathematical model has been used to investigate the SRM method’s thermochemical conversion in PBMR using an external energy loading. A mathematical model is developed to simulate the energy transfer of the gaseous and solid phases and transport of chemical components coupled to the SRM method’s thermochemical kinetic model in PBMR. A computational algorithm using the FORTRAN 95 language has been elaborated by the authors to compute the results as in the model equations of this work. In Table 1 and Table 2, the geometrical characteristics from PBMR, catalytic bed’s properties, and operating conditions at the inlet from PBMR are shown.

In order to ensure good results of the SRM method on PBMR, the convergence criterion for all results of this work has been secured using the ratio between the new variable value and the old variable value according to the new variable value as follows.

(55) $$\left|\frac{\alpha_{\mathrm{new},i}^{k+1}-\alpha_{\mathrm{old},i}^{k}}{\alpha_{\mathrm{new},i}^{k+1}}\right|\leq 10^{-6}$$

After specifying the main geometrical characteristics from PBMR, the catalytic bed’s properties and operating conditions at the inlet from PBMR are shown in the above tables. Table 3 and Table 4 show the numerical values of kinetic constants, equilibrium adsorption constants, equilibrium constants, thermophysical parameters, and dispersion coefficients of chemical components i in the reaction zone from PBMR. The parameter values of Table 1, Table 2, Table 3 and Table 4 are used to feed the developed computational algorithm for this work.

### 3.2. Validation by Comparison against Published Data

#### Mole Fractions at the Outlet of FBR and PBMR

**Figure 2:**
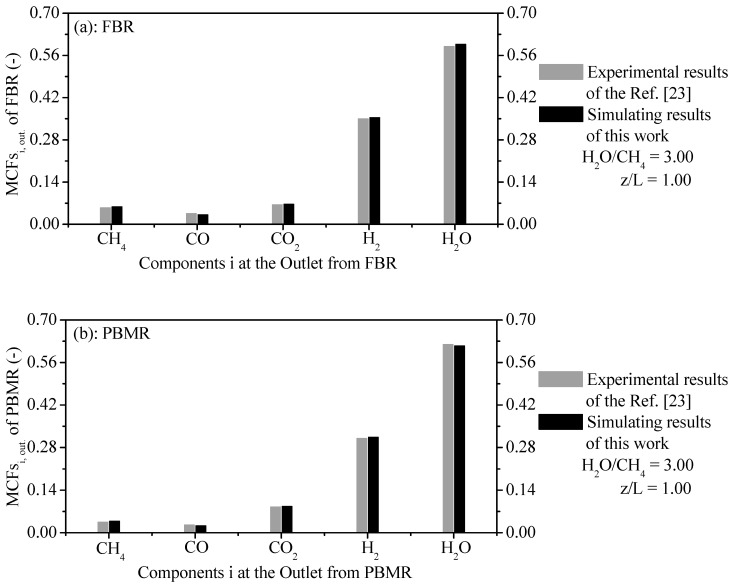
(**a**) Mole fractions of chemical components i at the outlet from Fixed Bed Reformer (FBR); (**b**) Mole fractions of chemical components i at the reaction zone’s outlet from PBMR.

To ensure the validity of the proposed model, authors have made a comparison of simulated results (SRs) against published data in the open literature. The SRs are computed from a developed computer code by authors. Figure 2 compares the literature results and simulating results of MFCs i at the outlet of the FBR and PBMR. Equation (56) has been used to compute the MFCs i values at the outlet of the reaction zone from FBR and PBMR.

(56) $${\mathrm{MFCs}}_{i,\mathrm{out}.}\;\mathrm{of}\;\mathrm{FBR}\;\mathrm{and}\;\mathrm{FBMR}=\frac{RT_{\mathrm{op}.}}{q_{g}P_{\mathrm{op}.}}F_{i};\;i={\mathrm{CH}}_{4},\;H_{2}O,\;H_{2},\;\mathrm{CO}\;\mathrm{and}\;{\mathrm{CO}}_{2}$$

The SRs for these two cases of the MFCs i are in agreement good with the literature data available in Ref. [23]. Slight differences can be found due to the deviation between the literature results and simulating results. An average relative error (ARE), Equation (57), was used to compute the consistency criterion between the results obtained and this ARE is given as follows.

(57) $$\mathrm{ARE}=\left|\frac{{\mathrm{MFCs}}_{i}^{\mathrm{Ref}.[23]}-{\mathrm{MFCs}}_{i}^{\mathrm{SRs}.}}{{\mathrm{MFCs}}_{i}^{\mathrm{Ref}.[23]}}\right|\times 100;\;i={\mathrm{CH}}_{4},\;H_{2}O,\;H_{2},\;\mathrm{CO}\;\mathrm{and}\;{\mathrm{CO}}_{2}$$

The good accordance between the SRs and literature data show that the developed model is acceptable. Considering the studied cases in Figure 2a,b, we obtained consistent satisfactory results from MFCs i against experimental results from the literature [23], resulting in AREs of $7.35\%\leq {\mathrm{ARE}}_{{\mathrm{CH}}_{4}}\leq 11.25\%$, $7.79\%\leq {\mathrm{ARE}}_{H_{2}O}\leq 11.68\%$, $1.85\%\leq {\mathrm{ARE}}_{H_{2}}\leq 4.07\%$, $0.65\%\leq {\mathrm{ARE}}_{\mathrm{CO}}\leq 3.23\%$, and $1.13\%\leq {\mathrm{ARE}}_{{\mathrm{CO}}_{2}}\leq 3.75\%$ to FBR and $5.03\%\leq {\mathrm{ARE}}_{{\mathrm{CH}}_{4}}\leq 8.05\%$, $6.71\leq {\mathrm{ARE}}_{H_{2}O}\leq 9.73\%$, $1.12\leq {\mathrm{ARE}}_{H_{2}}\leq 4.11\%$, $0.36\%\leq {\mathrm{ARE}}_{\mathrm{CO}}\leq 2.64\%$ and $0.31\%\leq {\mathrm{ARE}}_{{\mathrm{CO}}_{2}}\leq 2.69\%$ for PBMR.

The CIEA method has been used to simulate the results of the SRM method in PBMR and FBR. The CIEA method can be considered as a potential candidate for solving an NPDE system at lower CPU time. Table 5 shows the results of the SRM method in PBMR and FBR and, thus, these results are compared with the Finite Volume (FV) method against the CIEA method.

### 3.3. Simulating Process

#### 3.3.1. Temperature Profiles of Endothermic Reactions

**Figure 3:**
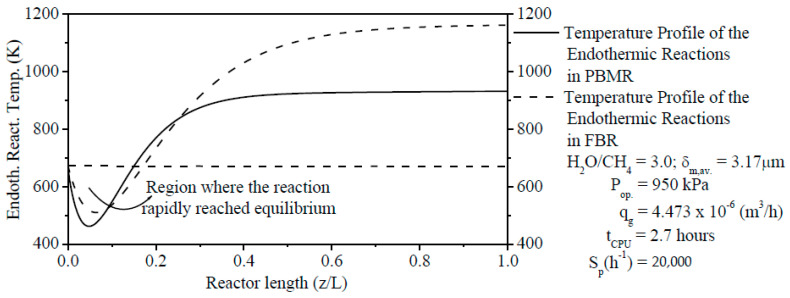
Comparison of the Temperature Profiles of Endothermic Reactions (TPERs) along the length of FBR and along the reaction zone length from PBMR on Ni/γ-Al_2_O_3_.

The Temperature Profiles of Endothermic Reactions (TPERs) have been computed inside FBR and within the reaction zone from PBMR and can be seen in Figure 3. It was shown that TPERs tend to assume inflection points of minimum values at which the SRM method’s minimum temperatures are found due to the effect of endothermic reactions. The location of the minimum values of these TPERs could be due to the interaction of many factors as the catalytic bed’s composition of the reaction zone from PBMR, initial temperature, operating pressure, and the thermodynamic equilibrium of endothermic reactions. It is clearly shown that the ERT of the FBR is much higher than the ERT in the reaction zone from PBMR. As it was reported in Figure 3, the TPER in PBMR’s reaction zone reached the stable state (at about z/L = ±0.5) faster than FBR. After achieving the stable state, ERT is kept constant up to z/L = 1.0 for FMBR. On the other hand, the TPER in FBR achieved the stable state at about z/L = ±0.85. As an advantage from PBMR compared to FBR, the thermodynamic equilibrium of reforming reactions in PBMR is obtained for a lesser reaction temperature due to the removal of H_2_ through Pd-based dense membrane.

#### 3.3.2. Effect of H_2_O/CH_4_ Ratio on the ERT

**Figure 4:**
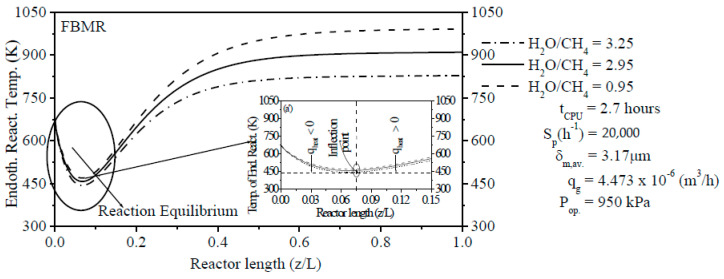
Effect of the H_2_O/CH_4_ ratio on the ERT along the reaction zone length from PBMR on Ni/γ-Al_2_O_3_.

Figure 4 describes the PBMR’s inlet H_2_O/CH_4_ ratio as an important parameter on the ERT inside reaction zone from PBMR. For PBMR, the decrease of the H_2_O/CH_4_ ratio has a negative effect on the ERT within the reaction zone. Incidentally, the main objective of PBMR is to carry out the thermochemical conversion of CH_4_ at moderate temperature because of the shift of the thermodynamic equilibrium on account of the removal of H_2_ through the membrane. Different values of the H_2_O/CH_4_ ratio were chosen for the comparison. When the H_2_O/CH_4_ ratio at the inlet from PBMR is low (H_2_O/CH_4_ = 0.95), the heat absorption in reaction zone section is notably increased and therefore, the ERT is favored.

On the other hand, when the H_2_O/CH_4_ ratio at the inlet is high (H_2_O/CH_4_ = 3.25), the heat absorption in the reaction zone is remarkably decreased and, thus, the ERT is reduced. It is clearly shown that the ERT attains the stable state at about z/L = ±0.70 and only a part of the reformer is utilized. After reaching the stable state, ERTs are maintained constant up to z/L = 1.0 in the reaction zone from PBMR with values of 828.49 K (H_2_O/CH_4_ = 3.25), 910.27 K (H_2_O/CH_4_ = 2.95), and 991.68 K (H_2_O/CH_4_ = 0.95), respectively. Figure 4 shows a symmetry region in relation to inflection points of TPERs. Before achieving the inflection points, there is a decrease of the heat rate (q_heat_ ˂ 0) due to the heat consumption for reforming reactions. After passing the inflection points, there is an increase of the heat rate (q_heat_ > 0) to keep the thermodynamic equilibrium of reforming reactions.

#### 3.3.3. Effect of the S_sp_ on the ERT

**Figure 5:**
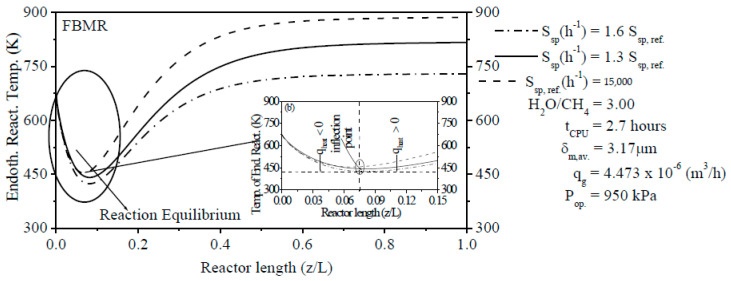
Effect of the S_sp_ on the ERT along the reaction zone length from PBMR on Ni/γ-Al_2_O_3_.

After investigating the H_2_O/CH_4_ ratio’s effect on the ERT, authors also studied the effect of the S_sp_ on the ERT inside reaction zone from PBMR. The S_sp_ is inversely proportional to the residence time. Thus, an increase of the S_sp_ indicates a decrease in the residence time of reactants within reaction zone from PBMR and, therefore, a reduction in the thermochemical conversion of CH_4_. In contrast, a decrease of the S_sp_ indicates an increase of the residence time (higher contact time between catalyst and reactants) of reactants in reaction zone from PBMR and, thus, a rise in the thermochemical conversion of CH_4_. As a result, Figure 5 shows that the reduction of the S_sp_ increases the ERT. An increase of the ERT has a positive effect on the thermochemical conversion of CH_4_ and yield of H_2_, i.e., the conversion of CH_4_ and yield of H_2_ increase with the rise of the ERT [3]. As it was analyzed in in Figure 4, three different values of the S_sp_ were used to check the sensibility of the ERT to the S_sp_. Figure 5 reports that the S_sp_ is inversely proportional to the ERT, i.e., when the S_sp_ is lower (S_sp,ref._ = 15,000 h^−1^), the ERT is notably increased. In contrast, when S_sp_ is higher (S_sp_ = 1.6 S_sp,ref_ = 24,000 h^−1^), the ERT is remarkably reduced. After achieving the stable state (z/L = ±0.70), ERTs are kept constant in reaction zone up to z/L = 1.0 from PBMR with values of 729.35 K (S_sp_ = 24,000 h^−1^), 816.47 K (S_sp_ = 19,500 h^−1^), and 885.98 K (S_sp,ref._ = 15,000 h^−1^), respectively.

#### 3.3.4. Distribution of Chemical Components i in PBMR

**Figure 6:**
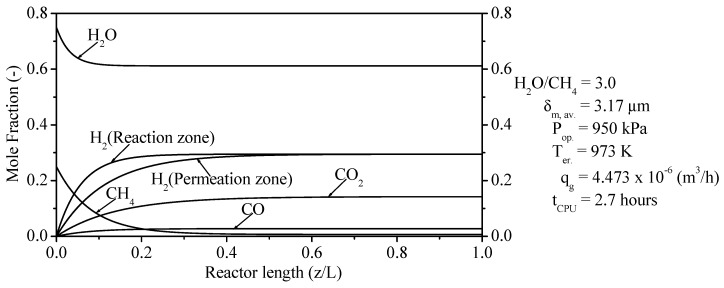
Molar fractions of components i along the reaction zone length from PBMR on Ni/γ-Al_2_O_3._

Figure 6 investigates the profiles of mole fractions for each component i of the SRM method in reaction zone and permeation zone from PBMR at the following operating conditions: H_2_O/CH_4_ = 3.00, 950 kPa, 973 K, and 4.473 × 10^−6^ (m^3^/h). In this figure, the profiles of mole fractions from consumed reactants (H_2_O and CH_4_) and produced products (CO, CO_2_ and H_2_) have been reported in the reaction zone as well as the profile of mole fraction of H_2_ in the permeation zone. H_2_O was not fully consumed for the SRM method within the reaction zone from PBMR, i.e., after reaching a stable consumption (z/L = ±0.17), only 18.67% was spent. On the other hand, CH_4_ was completely consumed for the SRM method inside the reaction zone from PBMR, after achieving a stable consumption (z/L = ±0.37), an amount of 97.26% was consumed. After reaching the stable mole fractions (z/L = ±0.52) of H_2_ in reaction zone and permeation zone, we obtained quantities of 29.49% (reaction zone) and 29.48% (permeation zone), respectively. Similarly, after attaining the stable mole fractions of CO_2_ (z/L = ±0.57) and CO (z/L = ±0.4) in the reaction zone, we computed the amounts of 14.23% and 2.76%, respectively.

#### 3.3.5. Conversion Rate of CH_4_ and Feed-Based Yield of H_2_ in FBR and FDMR

Thermodynamic limitations of FBRs are considered as a great problem to increase the thermochemical conversion of the SRM method. To solve this gap, we used PBMRs as innovating equipment that act with permselective membranes to overcome thermodynamic limitations and thus get a high conversion rate of CH_4_ at lower temperature [24]. The thermochemical performance of FBR and PBMR is analyzed from the Conversion Rate (CR) of CH_4_, and Feed-Based Yield (FBY) of H_2_ as follows.

(58) $$\mathrm{Conversion}\;\mathrm{Rate}\;(\mathrm{CR})\;\mathrm{of}\;{\mathrm{CH}}_{4}=1-\frac{F_{{\mathrm{CH}}_{4},\mathrm{out}}}{F_{{\mathrm{CH}}_{4},0}}$$

(59) $$\mathrm{Feed}-\mathrm{based}\;\mathrm{yield}\;(\mathrm{FBY})\;\mathrm{of}\;H_{2}=\frac{F_{H_{2},\mathrm{out}.}-F_{H_{2},\mathrm{in}.}}{F_{H_{2}O,\mathrm{in}.}+2F_{{\mathrm{CH}}_{4},\mathrm{in}.}}$$

**Figure 7:**
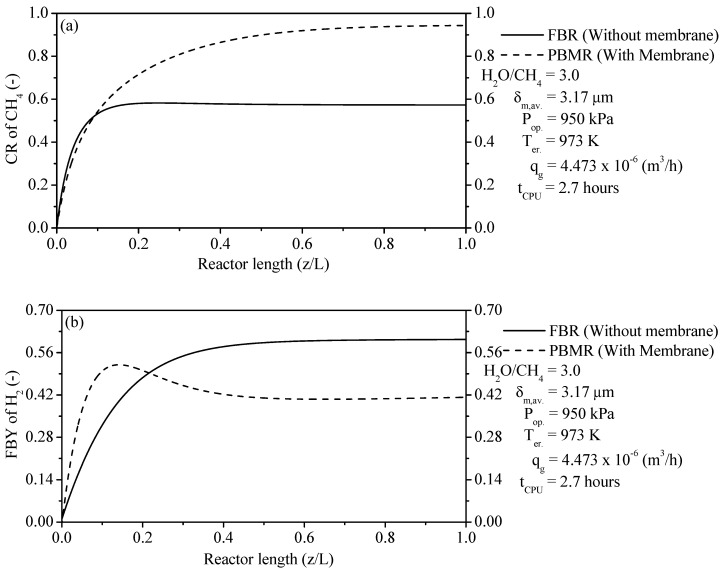
(**a**) Conversion Rate (CR) of CH_4_ for FBR and CR of CH_4_ in reaction zone from PBMR; (**b**) Feed-Based Yield (FBY) of H_2_ for FBR and FBY of H_2_ in reaction zone from PBMR.

Figure 7a compares the CR of CH_4_ inside FBR and CR of CH_4_ in reaction zone from PBMR. It is clearly shown that the thermochemical CR rate of CH_4_ in FBR is lower while a substantial improvement in the thermochemical CR of CH_4_ is achieved by PBMR. As a result, after achieving the stable state at about z/L = ±0.20 (see Figure 7a), the CR of CH_4_ is kept constant until z/L = 1.0 of FBR with a value of ±0.58 at the operating conditions of H_2_O/CH_4_ = 3.00, 950 kPa, 973 K, and 4.473 × 10^−6^ m^3^/h. A higher CR of CH_4_ in reaction zone from PBMR is favored by the shift in the thermodynamic equilibrium according to LeChatelier’s principle, sweep gas flux, change of concentration, operating temperature, and reduction of the partial pressure of H_2_ in the separation side. On the other hand, it is clearly shown that the CR of CH_4_ in reaction zone from PBMR is remarkably enhanced. After reaching the stable state at about z/L = ±0.80 (see Figure 7a), the CR of CH_4_ is maintained constant up to z/L = 1.0 in reaction zone from PBMR with a value of ±0.95 at the same operating conditions of FBR.

Figure 7b confronts the FBY of H_2_ within FBR and FBY of H_2_ in reaction zone from PBMR. It can be seen that the profiles of the FBY of H_2_ in two studied reformers have different behavior. As a result, the FBY of H_2_ for FBR rose notably until reaching a stable state. After that, it was kept constant up to z/L = 1.0 with a value of ±0.61 at the same operating conditions from Figure 7a. On the other hand, the FBY of H_2_ for PBMR tends to take on an inflection point of a maximum value at which the effective FBY of H_2_ is optimum. After attaining the maximum value at z/L = ±0.1, the FBY of H_2_ decreases up to z/L = ±0.50 and after that, it is maintained until z/L = 1.0 with a value of ±0.41 at the same operating conditions from Figure 7a. The profile from the FBY of H_2_ reports this behavior due to the removal of H_2_ through the membrane into the permeation zone from PBMR.

#### 3.3.6. Effect of the S_sp_ on the CR of CH_4_

The process of reforming reactions of CH_4_ includes the adsorption of the reactant composition in the gaseous phase, desorption of mixture gas, and residence time of gaseous reactants.

**Figure 8:**
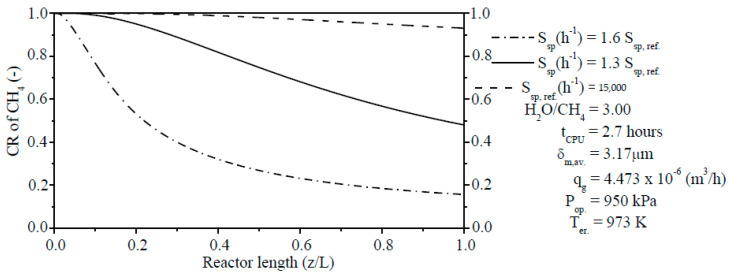
Effect of the S_sp_ on the CR of CH_4_ along the reaction zone length from PBMR on Ni/γ-Al_2_O_3_.

As the S_sp_ is inversely proportional to the residence time, an increase on the S_sp_ will reduce the residence time for the gaseous reactants on the catalyst particles. Conversely, a reduction of the S_sp_ points to an increase of the residence time of gaseous reactants to react on the catalyst particles and, thus, a rise in the CR of CH_4_. In order to understand the effect of the S_sp_ on the CR of CH_4_ (see Equation (58)), simulations are carried out for different S_sp_ values (S_sp,ref._ = 15,000 h^−1^, S_sp_ = 1.3 S_sp,ref._ = 19,500 h^−1^, and S_sp_ = 1.6 S_sp,ref._ = 24,000 h^−1^). As it can be seen in Figure 8, the CR of CH_4_ is reduced to be at z/L = 1.0 with the values of 16.32% (S_sp_ = 24,000 h^−1^), 48.28% (S_sp_ = 19,500 h^−1^), and 93.21% (S_sp,ref._ = 15,000 h^−1^), respectively.

#### 3.3.7. Effect of H_2_O/CH_4_ Ratio on the CR of CH_4_ and Consumption-Based Yield of H_2_

**Figure 9:**
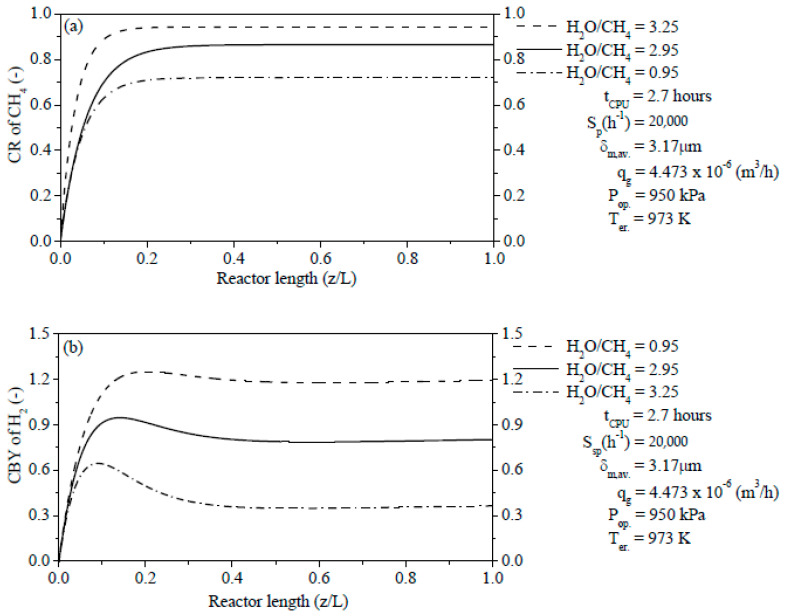
(**a**) Effect of the H_2_O/CH_4_ ratio on the CR of CH_4_ along of the reaction zone length from PBMR; (**b**) Effect of the H_2_O/CH_4_ ratio on the CBY of H_2_ along of the reaction zone length from PBMR.

The effect of the H_2_O/CH_4_ ratio plays a very significant function in the CR of CH_4_ and CBY of H_2_ in the reaction zone from PBMR. The results of the CR of CH_4_ in Figure 9a have been computed using Equation (58) from the above item. On the other hand, results of the CBY of H_2_ in Figure 9b were obtained through Equation (60) as follows.

(60) $$\mathrm{Consumption}-\mathrm{based}\;\mathrm{yield}\;(\mathrm{CBY})\;\mathrm{of}\;H_{2}=\frac{F_{H_{2},\mathrm{out}.}-F_{H_{2},\mathrm{in}.}}{\left(F_{H_{2}O,\mathrm{in}.}+2F_{{\mathrm{CH}}_{4},\mathrm{in}.}\right)-\left(F_{H_{2}O,\mathrm{out}.}+2F_{{\mathrm{CH}}_{4},\mathrm{out}.}\right)}$$

Figure 9a shows the profiles of the CR of CH_4_ at three different values of the H_2_O/CH_4_ ratio in reaction zone from PBMR. The CR of CH_4_ can be notably reduced with the increase of the coke formation on Ni catalysts. Based on the profiles from Figure 9a, it has been noted that an increase of the H_2_O/CH_4_ ratio raises the CR of CH_4_ due to the reduction of the coke content on the external surface of the catalyst particles. On the other hand, a decrease of the H_2_O/CH_4_ ratio reduces the CR of CH_4_ owing to the increase of the coke formation on the outside surface of catalyst particles. As a result, the CR of CH_4_ was reduced to be at z/L = 1.0 with the values of 94.75% (H_2_O/CH_4_ = 3.25), 86.57% (H_2_O/CH_4_ = 2.95), and 72.06% (H_2_O/CH_4_ = 0.95), respectively.

Figure 9b reports the profiles of the CBY of H_2_ (Equation (60)) at three different values of the H_2_O/CH_4_ ratio in reaction zone from PBMR. Unlike of the CR of CH_4_, the profiles of the CBY of H_2_ sharply decrease with the increase of the H_2_O/CH_4_ ratio. An increase of steam content has a negative effect on the production of H_2_, i.e., the generating rate of H_2_ isn’t strengthened according to Figure 9b. The profiles of the CBY of H_2_ tend to take on inflection points of maximum values at which the effective CBYs of H_2_ is optimum. After achieving the maximum values, the CBYs of H_2_ decrease and after that, they are kept up to z/L = 1.0 with values of 1.1958 (H_2_O/CH_4_ = 0.95), 0.8169 (H_2_O/CH_4_ = 2.95), and 0.3572 (H_2_O/CH_4_ = 3.25), respectively.

#### 3.3.8. Thermochemical Energy Storage Efficiency

The reforming reaction’s thermochemical conversion of CH_4_ is a new technology which provides the advantage of high storage densities and minor thermal losses. The η_teses_ in PBMRs using the SRM method play an important role in energy storage and this will be discussed in this section. The η_tese_ of the SRM process in PBMR is computed as the ratio between the net chemical energy produced per the input electric power as follows.

(61) $$\eta_{\mathrm{tese}}\left(\%\right)=\frac{Q_{\mathrm{che}.}}{P_{\mathrm{elet}.}}=\frac{\rho_{s}\frac{\left(1-\varepsilon_{P}\right)}{\varepsilon_{P}}\sum_{j=1}^{3}\left(\pm \Delta H_{j}\right)\eta_{j}R_{j}}{U_{\mathrm{ghe}}S_{\mathrm{she}}\left(T_{\mathrm{er}}-T_{g}\right)}$$

**Figure 10:**
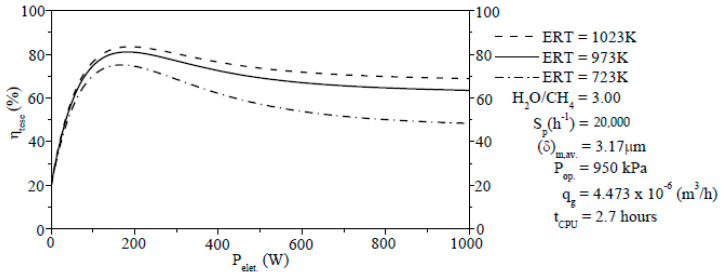
Effect of the ERT on the η_tese_ of the SRM method in PBMR on Ni/Mg/γ-Aℓ_2_O_3_ with 6% Ni loading.

Figure 10 describes the curves of the η_tese_ (Equation (61)) at three different values of the ERT in reaction zone from PBMR. A raise of the ERT has a positive effect on the η_tese_, i.e., the η_tese_ is improved according to Figure 10. As a result, it is clearly noted that an increase of the ERT will lead to higher η_teses_. The curves of η_teses_ trend to assume inflection points of maximum values (P_elet._ = ±150 W) in which the effective η_tese_ are optimum. After reaching the maximum values, the η_teses_ decrease and then they are maintained until P_elet._ = 1000 W with values of 68.96% (ERT = 1023 K), 63.21% (ERT = 973 K), and 48.12% (ERT = 723 K), respectively.

#### 3.3.9. Energy Storage Performance

**Figure 11:**
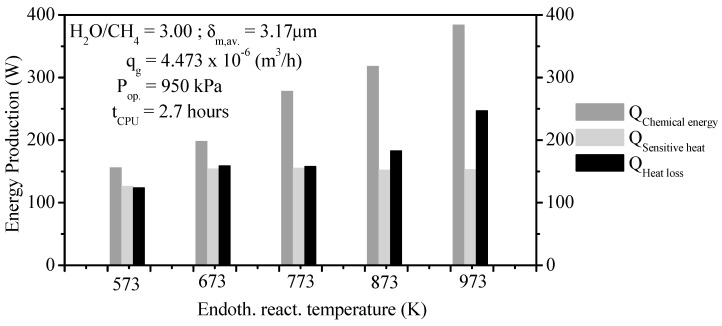
Energy storage performance of the thermochemical system as a function of the ERT.

The energy storage’s importance has motivated researchers of this work to study the energetic aspects of the storing technology as from thermochemical conversion. The chemical energy storage, sensible heat, and heat loss play important roles in the energy storage process. Figure 11 shows the energy storage performances of the thermochemical reforming method of CH_4_ for different reaction temperatures in reaction zone from PBMR. As it can be seen in this figure, as the ERT increases the chemical energy and the heat loss increase drastically. On the other hand, the sensible heat gradually increases as the ERT rises. After reaching the ERT of 973 K, the chemical energy, sensible heat, and heat loss had values of 384.96 W, 151.68 W, and 249.73 W, respectively.

#### 3.3.10. Selectivity of Components of the SRM Method

The loading process of thermal energy on PBMR is used to drive the endothermic reactions of CH_4_. The thermal energy is thermochemically employed to convert reactants (CH_4_ and H_2_O) into products (H_2_, CO, and CO_2_). As a result, the performance of components (CH_4_, H_2_, CO, and CO_2_) of the SRM method were analyzed in terms of the CR of CH_4_, FBY of H_2_, CBY of H_2_, and selectivity of H_2_, CO, CO_2_ and CH_4_. A set of corresponding expressions are used to compute the selectivity of H_2_, CO, CO_2_, and CH_4_ as follows.

(62) $$S_{H_{2}}\left(\%\right)=\frac{\left(F_{H_{2},\mathrm{rz}}^{\mathrm{out}.}+F_{H_{2},\mathrm{pz}}^{\mathrm{out}.}\right)-F_{H_{2}}^{\mathrm{in}.}}{\left(F_{H_{2},\mathrm{rz}}^{\mathrm{out}.}+F_{H_{2},\mathrm{pz}}^{\mathrm{out}.}\right)+\left(F_{\mathrm{CO},\mathrm{rz}}^{\mathrm{out}.}+F_{{\mathrm{CO}}_{2},\mathrm{rz}}^{\mathrm{out}.}+F_{{\mathrm{CH}}_{4},\mathrm{rz}}^{\mathrm{out}.}\right)}\times 100$$

(63) $$S_{\mathrm{CO}}\left(\%\right)=\frac{F_{\mathrm{CO},\mathrm{rz}}^{\mathrm{out}}-F_{\mathrm{CO},\mathrm{rz}}^{\mathrm{in}.}}{\left(F_{H_{2},\mathrm{rz}}^{\mathrm{out}.}+F_{H_{2},\mathrm{pz}}^{\mathrm{out}.}\right)+\left(F_{\mathrm{CO},\mathrm{rz}}^{\mathrm{out}.}+F_{{\mathrm{CO}}_{2},\mathrm{rz}}^{\mathrm{out}.}+F_{{\mathrm{CH}}_{4},\mathrm{rz}}^{\mathrm{out}.}\right)}\times 100$$

(64) $$S_{{\mathrm{CO}}_{2}}\left(\%\right)=\frac{F_{{\mathrm{CO}}_{2},\mathrm{rz}}^{\mathrm{out}.}-F_{{\mathrm{CO}}_{2},\mathrm{rz}}^{\mathrm{in}.}}{\left(F_{H_{2},\mathrm{rz}}^{\mathrm{out}.}+F_{H_{2},\mathrm{pz}}^{\mathrm{out}.}\right)+\left(F_{\mathrm{CO},\mathrm{rz}}^{\mathrm{out}.}+F_{{\mathrm{CO}}_{2},\mathrm{rz}}^{\mathrm{out}.}+F_{{\mathrm{CH}}_{4},\mathrm{rz}}^{\mathrm{out}.}\right)}\times 100$$

(65) $$S_{{\mathrm{CH}}_{4}}\left(\%\right)=\frac{F_{{\mathrm{CH}}_{4},\mathrm{rz}}^{\mathrm{out}.}-F_{{\mathrm{CH}}_{4},\mathrm{rz}}^{\mathrm{in}.}}{\left(F_{H_{2},\mathrm{rz}}^{\mathrm{out}.}+F_{H_{2},\mathrm{pz}}^{\mathrm{out}.}\right)+\left(F_{\mathrm{CO},\mathrm{rz}}^{\mathrm{out}.}+F_{{\mathrm{CO}}_{2},\mathrm{rz}}^{\mathrm{out}.}+F_{{\mathrm{CH}}_{4},\mathrm{rz}}^{\mathrm{out}.}\right)}\times 100$$

Table 6 shows the effect of the dense membrane thickness (δ_m_) on the selectivity of chemical components (H_2_, CO, CO_2_, and CH_4_) in PBMR. For each δ_m_, we noted a different value of the selectivity for each chemical component at same operating conditions: H_2_O/CH_4_ = 3.00, 950 kPa, 973 K, and 4.473 × 10^−6^ m^3^/h. As a result, H_2_ presented higher values of the selectivity varying from 60.98% to 73.18% while CH_4_ reported lower values of the selectivity ranging from 1.41% to 2.06%. On the other hand, CO presented corresponding values of the selectivity from 10.71% to 15.15%, whilst CO_2_ has related relative values of the selectivity ranging from 14.69% to 21.37%.

## 4. Conclusions and Future Work

The present work has been focused on a numerical analysis of physical-mathematical modelling and computer simulation to describe the performance of reformers for the production of H_2_ using a reference method of steam reforming CH_4_. The model equations that describe the gas temperature in the reaction zone, endothermic reaction’s temperature in the reaction zone, molar flow of the components i in the reaction zone, molar flow of H_2_ in the reaction zone, and molar flow of H_2_ in the permeation zone have been reported and discussed. As a solution to the model equations, the main focus has been the CIEA method as a powerful technique to reduce the NPDE system of this work into a NODE system using the boundary conditions of each NPDE. The work’s results highlighted the importance of the mathematical model developed to describe the performance from FBR and PBMR. In this context, the main conclusions are summarized as follows.

1. The computed results of MFCs are compared with experimental results from the literature under same operating conditions for FBR and PBMR. Figure 2a compares the computed results of MFCs against experimental results of MFCs of components CH_4_, CO, CO_2_, H_2_, and H_2_O at the outlet from FBR with AREs around $7.35\%\leq {\mathrm{ARE}}_{{\mathrm{CH}}_{4}}\leq 11.25\%$, $7.79\%\leq {\mathrm{ARE}}_{H_{2}O}\leq 11.68\%$, $1.85\%\leq {\mathrm{ARE}}_{H_{2}}\leq 4.07\%,$ $0.65\%\leq {\mathrm{ARE}}_{\mathrm{CO}}\leq 3.23\%,$ and $1.13\%\leq {\mathrm{ARE}}_{{\mathrm{CO}}_{2}}\leq 3.75\%,$ respectively. On the other hand, Figure 2b also confronts the simulated results of MFCs against the experimental results of the MFCs of components CH_4_, CO, CO_2_, H_2_, and H_2_O at the outlet from FMBR with AREs of about $5.03\%\leq {\mathrm{ARE}}_{{\mathrm{CH}}_{4}}\leq 8.05\%$, $6.71\leq {\mathrm{ARE}}_{H_{2}O}\leq 9.73\%$, $1.12\leq {\mathrm{ARE}}_{H_{2}}\leq 4.11\%$, $0.36\%\leq {\mathrm{ARE}}_{\mathrm{CO}}\leq 2.64\%$ and $0.31\%\leq {\mathrm{ARE}}_{{\mathrm{CO}}_{2}}\leq 2.69\%$, respectively.
2. The thermochemical conversion of CH_4_ and production of H_2_ are considered the two main parameters of FBR and PBMR. The ERT has a positive effect on these two parameters. When comparing the two reformer types, the production of H_2_ and thermochemical conversion of CH_4_ on the PBMR can be obtained at lower ERT due to the removal of H_2_ through a membrane from PBMR. This point is a great advantage from PBMR in relation to FBR because PBMR can be operated at lower ERT than FBR and to obtain higher results of these two parameters.
3. The steam content has an important effect on the ERT in PBMR, i.e., when the H_2_O/CH_4_ ratio is low, the ERT is notably increased. In contrast, when the H_2_O/CH_4_ ratio is high, the ERT is remarkably decreased. The S_sp_ has also a significant effect on the ERT and CR of CH_4_. Similar to steam content, when the S_sp_ is small, the ERT is remarkably increased. On the other hand, the ERT is reduced with the increase of the S_sp_. For a lower Ssp value, we observed a higher CR of CH_4_ in PBMR. Conversely, a higher S_sp_ value points to a low CR of CH_4_ in PBMR.
4. The effect of the H_2_O/CH_4_ ratio played a significant role on the CR of CH_4_ and CBY of H_2_. An increase of the H_2_O/CH_4_ ratio is associated with a significant improvement due to the coke reduction on the catalytic particles, i.e., there is a rise of the CR of CH_4_. Conversely, a decrease of the H_2_O/CH_4_ ratio has a poor effect owing to the increase of the coke formation and thus the CR of CH_4_ is reduced. Unlike the CR of CH4, a rise of the H_2_O/CH_4_ ratio led to a decrease of the CBY of H_2_. On the other hand, a reduction of the H_2_O/CH_4_ ratio led to an increase of the CBY of H_2_.
5. An increase of the ERT led to a rise of the η_tese_. Meanwhile, a reduction of ERT resulted in an decrease of the η_tese_. A rise of the ERT has a positive effect on the chemical storage, i.e., the chemical energy gradually rose with the increase of ERT (156.01 W (573 K)–384.96 W (973 K)). The selectivity of components (H_2_, CO, CO_2_, and CH_4_) was computed for different δ_m_. Among these components, H_2_ showed higher amounts (60.98% to 73.18%) of the selectivity.

In future research, the exploration of novel materials like open-cell foams can be explored in the context of reforming reactions. Solid open-cell foams constitute a class of porous materials with low density and improved thermal properties. In addition, open-cell foams can be considered as potential candidates for catalyst support in the gas-solid reaction field due to their high external surface area, high porosity, and low drop pressure. Thus, solid open-cell foams are future trends for reforming methods such as steam reforming of CH_4_, dry reforming of CH_4_, etc.

## Figures and Tables

**Table 1:** PBMR’s Geometrical characteristics and the catalytic bed’s properties.

	PBMR	Sources
**Geometrical characteristics**		
L (m)	0.18	Estimated
r_rz_ (m)	0.0020	Ref. [3]
r_1_ (m)	0.0010	Ref. [3]
r_2_ (m)	0.0015	Ref. [3]
r_3_ (m)	0.035	Ref. [3]
δ_m,av._ (μm)	3.17	Estimated
**Properties of the catalyst bed**		
ε_b_ (m^3^gas/m^3^reformer)	0.39	Ref. [3]
ε_p_ (m^3^gas/m^3^reformer)	0.43	Ref. [3]
η_1_ (Equation (5))	0.0121	Ref. [3]
η_2_ (Equation (6))	0.0169	Ref. [3]
η_3_ (Equation (7))	0.0191	Ref. [3]
d_p_ (m)	79 μm	Estimated
$\rho_{s}\left(\mathrm{kg}/m^{3}\right)$	2.500	Ref. [6]
S_p_ (h^−1^)	0.796	Ref. [6]
M_bed_ (kg)	9.56 × 10^−3^	Estimated

**Table 2:** Operating conditions at the inlet from PBMR.

	PBMR	Sources
**Operating conditions**		
q_g_ (m^3^/h)	4.473 × 10^−6^	Ref. [6]
V_sg_ (m/h)	3.34 × 10^−2^	Ref. [6]
g (m/h^2^)	1.271 × 10^8^	Ref. [6]
ρ_g_ (k/m^3^)	0.1692	Ref. [6]
$Q_{0}\left(\mathrm{kmol}/{m\;\mathrm{kPa}}^{0.5}h\right)$	7.173 × 10^−5^	Ref. [6]
$E_{p}\left({\mathrm{kPa}\;m}^{3}/\mathrm{kmol}\right)$	5.576 × 10^4^	Ref. [6]
$R\left({\mathrm{kPa}\;m}^{3}/\mathrm{kmol}\;K\right)$	8.314	Ref. [6]
T_g,0_ (K)	673	Ref. [6]
T_er,0_ (K)	673	Ref. [6]
P_op._ (kPa)	950	Ref. [3]
P_op,per_ (kPa)	150	Ref. [3]
$F_{{\mathrm{CH}}_{4},\mathrm{in}.}\left(\mathrm{kmol}|h\right)$	0.282	Ref. [3]
$F_{H_{2}O,\mathrm{in}.}\left(\mathrm{kmol}|h\right)$	0.602	Ref. [3]
$F_{H_{2},\mathrm{in}.}\left(\mathrm{kmol}|h\right)$	0.076	Ref. [3]
$F_{\mathrm{CO},\mathrm{in}.}\left(\mathrm{kmol}|h\right)$	0.032	Ref. [3]
$F_{{\mathrm{CO}}_{2},\mathrm{in}.}\left(\mathrm{kmol}|h\right)$	6.32 × 10^−5^	Ref. [3]
$F_{\mathrm{sweep}}\left(\mathrm{kmol}|h\right)$	5$F_{{\mathrm{CH}}_{4},\mathrm{in}.}$	Ref. [3]

**Table 3:** Numeric values of kinetic constants, adsorption constants, and equilibrium constants for simulating of the SRM method on PBMR.

	Model Parameters	Sources
**Kinetic constants**		
$k_{1}\left({\mathrm{kmol}\;\mathrm{kPa}}^{0.5}/{\mathrm{kg}}_{\mathrm{cat}.}h\right)$	7.215 × 10^14^	Ref. [3]
$k_{2}\left({\mathrm{kmol}\;\mathrm{kPa}}^{-1}/{\mathrm{kg}}_{\mathrm{cat}.}h\right)$	1.218 × 10^3^	Ref. [3]
$k_{3}\left({\mathrm{kmol}\;\mathrm{kPa}}^{0.5}/{\mathrm{kg}}_{\mathrm{cat}.}h\right)$	9.701 × 10^15^	Ref. [3]
**Equilibrium adsorption constants**		
$K_{{\mathrm{CH}}_{4}}\left({\mathrm{kPa}}^{-1}\right)$	8.974 × 10^−7^	Ref. [3]
$K_{H_{2}O}\left(-\right)$	3.701 × 10^4^	Ref. [3]
$K_{H_{2}}\left({\mathrm{kPa}}^{-1}\right)$	8.987 × 10^−12^	Ref. [3]
$K_{\mathrm{CO}}\left({\mathrm{kPa}}^{-1}\right)$	5.671 × 10^−6^	Ref. [3]
**Equilibrium constants**		
$K_{\mathrm{eq},1}\left({\mathrm{kPa}}^{2}\right)$	2.135 × 10^7^	Ref. [3]
$K_{\mathrm{eq},2}\left(-\right)$	13.015	Ref. [3]
$K_{\mathrm{eq},3}\left({\mathrm{kPa}}^{2}\right)$	7.102 × 10^5^	Ref. [3]

**Table 4:** Numerical values of thermophysical parameters and dispersion coefficients of chemical components for simulating the SRM method in PBMR.

	Model Parameters	Sources
**Thermophysical parameters**		
$C_{p,g,i}\left(J/\mathrm{kg}\;K\right)$	0.987	Ref. [6]
$\lambda_{g,\mathrm{eff}}\left(W/m\;K\right)$	0.0185	Equation (25)
$h_{\mathrm{gs}}\left(W/m^{2}\;K\right)$	1.902	Ref. [6]
$C_{p,s}\left(J/\mathrm{kg}\;K\right)$	336	Ref. [6]
$\lambda_{s,\mathrm{eff}}\left(W/m\;K\right)$	0.0289	Equation (31)
${\Delta H}_{\left(700{}^{\circ}C\right)}^{\mathrm{Eq}.\left(5\right)}\left(\mathrm{kJ}/\mathrm{kmol}\right)$	281.83	Ref. [6]
${\Delta H}_{\left(700{}^{\circ}C\right)}^{\mathrm{Eq}.\left(6\right)}\left(\mathrm{kJ}/\mathrm{kmol}\right)$	−35.67	Ref. [6]
${\Delta H}_{\left(700{}^{\circ}C\right)}^{\mathrm{Eq}.\left(7\right)}\left(\mathrm{kJ}/\mathrm{kmol}\right)$	209.51	Ref. [6]
**Dispersion coeffs. of components i**		
$D_{\mathrm{ax},{\mathrm{CH}}_{4}}\left(m^{2}/h\right)$	0.0289	Ref. [6]
$D_{\mathrm{ax},H_{2}O}\left(m^{2}/h\right)$	0.0379	Ref. [6]
$D_{\mathrm{ax},H_{2}}\left(m^{2}/h\right)$	0.0201	Ref. [6]
$D_{\mathrm{ax},\mathrm{CO}}\left(m^{2}/h\right)$	0.0341	Ref. [6]
$D_{\mathrm{ax},{\mathrm{CO}}_{2}}\left(m^{2}/h\right)$	0.0189	Ref. [6]

**Table 5:** Comparison of the results between the Finite Volume (FV) method and Coupled Integral Equation Approach (CIEA) method.

	FV Method				CIEA Method			
	PBMR (t_CPU_ = 3.2 h)		FBR (t_CPU_ = 3.4 h)		PBMR (t_CPU_ = 2.5 h)		FBR (t_CPU_ = 2.6 h)	
Compts.	Ref. [23]	Teor.	Ref. [23]	Teor.	Ref. [23]	Teor.	Ref. [23]	Teor.
CH_4_	0.035	0.0382	0.055	0.0586	0.035	0.0386	0.055	0.0567
CO	0.026	0.0241	0.036	0.0323	0.026	0.0231	0.036	0.0319
CO_2_	0.085	0.0839	0.065	0.0667	0.085	0.0867	0.065	0.0670
H_2_	0.301	0.3101	0.352	0.3548	0.301	0.3142	0.352	0.3567
H_2_	0.621	0.6017	0.591	0.5971	0.621	0.6151	0.591	0.6001

**Table 6:** Selectivity of H_2_, CO, CO_2_, and CH_4_ as a function of the dense membrane thicknesses (δ_m_).

	PBMR (H_2_O/CH_4_ = 3.00, P_zr_ = 950 kPa, P_pz_ = 150 kPa, and ERT = 973 K)				
δ_m_ (μm)	1.7	2.7	3.7	4.7	5.7
Selectivities					
$S_{H_{2}}\left(\%\right)$	60.98	73.18	68.39	64.78	61.95
$S_{\mathrm{CO}}\left(\%\right)$	15.17	10.71	12.62	14.06	15.19
$S_{{\mathrm{CO}}_{2}}\left(\%\right)$	21.37	14.69	17.31	19.29	20.84
$S_{{\mathrm{CH}}_{4}}\left(\%\right)$	2.06	1.41	1.67	1.85	2.03

## Nomenclature

$C_{p,g,i}$	Heat capacity of components i (kJ/kg K)
$C_{p,g,\mathrm{mix}.}$	Molar heat capacity at constant pressure of the gas mixture (kJ/kg K)
$C_{p,s}$	Heat capacity pressure of the solid phase (kJ/kg K)
$D_{\mathrm{ax},i}$	Dispersion coefficients of each component i (m^2^/h)
$D_{\mathrm{ax},H_{2}}$	Dispersion coefficient of H_2_ (m^2^/h)
$d_{p}$	Diameter of the solid particles (m)
$d_{\mathrm{rz}}$	Diameter of the reaction zone (m)
$E_{H_{2}}$	Activation energy of H_2_ (kJ/kg)
$F_{i}$	Molar flow of components i (kmol/h)
$F_{j,0}$	Initial molar flows of components j for Equation (17), j = H_2_O, CO, CO_2_ and H_2_, (kmol/h)
$F_{H_{2}}$	Molar flow of H_2_ (kmol/h)
$F_{H_{2},\mathrm{per}.}$	Molar flow of H_2_ in the permeation zone (kmol/h)
g	Gravity acceleration (m/h^2^)
$h_{\mathrm{gs}}$	Gas-solid heat transfer coefficient (W/m^2^ K)
$i_{\mathrm{elet}.}$	Electric current (A)
$J_{H_{2},\mathrm{per}.}$	Permeation rate of H_2_ from the reaction zone into the permeation zone (kmol/m^2^h)
$k_{1}$	Reaction constant of the R_1_ (kmol kPa^0.5^/kgcat h)
$k_{2}$	Reaction constant of the R_2_ (kmol kPa^−1^/kgcat h)
$k_{3}$	Reaction constant of the R_3_ (kmol kPa^0.5^/kgcat h)
$K_{\mathrm{eq}.,1}$	Equilibrium constant of Equation (8), (kPa^2^)
$K_{\mathrm{eq}.,2}$	Equilibrium constant of Equation (9), (-)
$K_{\mathrm{eq}.,3}$	Equilibrium constant of Equation (10), (kPa^2^)
$k_{\mathrm{gs},\mathrm{eff}.}$	Effective gas-solid transfer (k_gs,eff_ = ε_b_ k_gs_), (m/h)
$K_{i}$	Adsorption constants of components i, i = CO, H_2_ and CH_4_, (kPa^−1^)
$K_{j}$	Adsorption constant of components j, j = H_2_O, (-)
L	PBMR length (m)
$P_{\mathrm{elet}.}$	Electric Power of the electric furnace (W)
P_i_	Partial pressures of components i, i = CH_4_, H_2_O, CO, CO_2_ and H_2_, (kPa)
P_op._	Operating pressure inside the reaction zone (kPa)
$P_{\mathrm{op},\mathrm{per}.}$	Operating pressure within the permeation zone (kPa)
$P_{H_{2},\mathrm{rz}}$	Partial pressure of H_2_ in the reaction zone (kPa^0.5^)
$P_{H_{2},\mathrm{per}.}$	Partial pressure of H_2_ in the permeation zone (kPa^0.5^)
$Q_{\mathrm{che}.}$	Chemical energy of the PBMR (W)
Q_0_	Pre-exponential factor of the Arrhenius law (kPa^0.5^/m h)
Q_eq._	Chemical energy storage as enthalpy (W)
$q_{g}$	Flow rate of the gas phase (m^3^/h)
$q_{h}$	Local heat flux (W/m^2^)
Q_Loss_	Heat loss from PBMR (W)
$Q_{\mathrm{sh}.}$	Sensible heat of the reaction products (W)
R	Electrical resistance (Ω)
$r_{i}$	Net rates of components i (i = CH_4_, H_2_O, CO, CO_2_ and H_2_) in PBMR (kmol/kg_cat._h)
$R_{j}$	Overall rates of the reforming reactions j, j = 1, 2 and 3, (kmol/kg_cat._h)
$r_{\mathrm{rz}}$	Inner radius of the reaction zone
S_sp_	Spatial velocity (h^−1^)
S_she_	Surface of heat exchange (m^2^)
t	Iteration time (h)
$T_{\mathrm{er},0}$	Initial temperature of the endothermic reaction (K)
$T_{\mathrm{er}}$	Temperature of the endothermic reaction (K)
$T_{g,0}$	Initial temperature of the gas phase (K)
$u_{\mathrm{sg}}$	Gas superficial velocity (m/h)
U_ghe_	Global heat exchange (W/m^2^ K)
z	Axial coordinate (m)
**Greek letters**	
$\alpha_{\mathrm{new},i}^{k+1}$	New variable
$\alpha_{\mathrm{old},i}^{k+1}$	Old variable
ΔH_j_	Enthalpy heat of the reactions (kJ/kmol)
δ_m_	Membrane thickness (m)
ε_b_	Fixed-bed porosity (m^3^gas/m^3^reformer)
$\varepsilon_{p}$	Particle porosity (m^3^particle/m^3^reformer)
η_j_	Effectiveness factors from the reforming reactions j (-)
$\lambda_{g,\mathrm{mix}.}$	Thermal conductivity of the gaseous mixture (W/m K)
$\lambda_{g,\mathrm{eff}}$	Effective thermal conductivity of the gas phase (W/m K)
$\lambda_{s,\mathrm{eff}}$	Effective solid thermal conductivity of the solid phase (W/m K)
ρ_g, mix._	Gas mixture density of components i (kg/m^3^)
ρ_s_	Solid phase density (kg/m^3^)
σ	Dimensionless parameter given by Equation (17), (-)
$\sigma_{\mathrm{ij}}$	Stiochiometric coefficients of the reforming reactions (-)
**Subscripts**	
0	Initial
ax	Axial
b	Bed
che.	Chemical Energy
elet.	Electric
eff	Effective
eq.	Equilibrium
er	Endothermic reaction
g	Gas phase
gs	Solid-gas
i	Chemical components
in.	Inlet
j	Reaction index
m	Membrane
mix.	Mixture
op.	Operating
out	Outlet
p	Particle
per.	Permeation
rz	Reaction zone
s	solid
sh.	Sensible heat
sp	Spatial

## Abbreviations

ARE	Average relative error
Bot.	Bottom
CR	Conversion rate
CBY	Consumption-based yield
CIEA	Coupled Integral Equation Approach
DRM	Dry reforming of methane
ERT	Endothermic reaction temperature
FBR	Fixed bed reformer
PBMR	Fixed bed membrane reformer
FBY	Food-based yield
K	Kelvin
MFC	Mole fractions of components
MRs	Membrane reformers
NIPHD	Non-isothermal pseudo-homogeneous dynamic
NPDE	Nonlinear Partial Differential Equation
NODE	Nonlinear Ordinary Differential Equation
SRM	Steam reforming of methane
SRs	Simulating results
TES	Thermochemical Energy Storage
TPER	Temperature profiles of endothermic reaction
Up.	Upper
W	Watt

## Appendix A

### Appendix A.1. Hermite Approximation

The key idea for the coupled integral equations approximation (CIEA) has been reported using the Hermite approach [7]. The mathematical model of this work is built by a system of NPDEs for the heat transfer and mass transfer of the SRM method in PBMR. The system of NPDEs is transformed into a system of NODEs applying the CIEA method taking into account the boundary conditions of each NPDE. Expressions were used to transform PDEs into NODEs with boundary conditions, and these expressions are described as follows.

(A1) $${\bar{f}}_{j}\left(t\right)=\frac{1}{L}\int_{0}^{L}f_{j}\left(z,t\right)\mathrm{dz};\;j=g,s,i$$

(A2) $$f_{j}\left(L,t\right)-f_{j}\left(0,t\right)≅\frac{L}{2}\left[{\frac{\partial f_{j}\left(z,t\right)}{\partial z}|}_{z=0}+{\frac{\partial f_{j}\left(z,t\right)}{\partial z}|}_{z=L}\right];\;j=g,s,i$$

(A3) $${\bar{f}}_{j}\left(t\right)≅\frac{L}{2}\left[f_{j}\left(0,t\right)+f_{j}\left(L_{z},t\right)\right]+\frac{L^{2}}{12}\left[{\frac{\partial f_{j}\left(z,t\right)}{\partial z}|}_{z=0}-{\frac{\partial f_{j}\left(z,t\right)}{\partial z}|}_{z=L}\right];\;j=g,s,i$$

### Appendix A.2. CIEA Method’s Application to the Energy Balance of the Gas Phase in Reaction Zone from PBMR

The boundary conditions (Equations (28) and (29)) of the energy balance of the gas phase at the inlet and outlet of the reaction zone from PBMR have been used to transform Equation (24) into Equation (47). The coefficients from Equation (47) as well as expressions of T_g_ (0, t) and T_g_ (L, t) are shown as follows.

(A4) $$\alpha_{g,1}=\rho_{g,\mathrm{mix}.}C_{p,g,\mathrm{mix}.};\;\alpha_{g,2}=\rho_{g,\mathrm{mix}.}C_{p,g,\mathrm{mix}.}\frac{4q_{g}}{\pi d_{\mathrm{rz}}^{2}}$$

(A5) $$\alpha_{g,3}=h_{\mathrm{gs}}\frac{\left(1-\varepsilon_{b}\right)}{\varepsilon_{b}}\frac{6}{d_{P}};\;\alpha_{g,4}=\frac{\rho_{g,\mathrm{mix}.}C_{p,g,\mathrm{mix}.}}{\lambda_{g,\mathrm{mix}.}};\;\alpha_{g,5}=\frac{h_{\mathrm{gs},\mathrm{eff}.}}{\lambda_{g,\mathrm{eff}.}}$$

(A6) $$\alpha_{g,6}=L\left(6+L\;\alpha_{g,4}\right)-\frac{L\left(6+L\;\alpha_{g,5}\right)\left(1+0.5L\;\alpha_{g,4}\right)}{1+0.5L\;\alpha_{g,5}}$$

(A7) $$\alpha_{g,7}\left(t\right)=12{\bar{T}}_{g}\left(t\right)+L^{2}\left(\alpha_{g,4}T_{g,\infty }^{\mathrm{Up}.}+\alpha_{g,5}T_{g,\infty }^{\mathrm{Bot}.}\right)\left[1+\frac{0.5\left(6+L\;\alpha_{g,5}\right)}{1-0.5L\;\alpha_{g,5}}\right]$$

(A8) $$T_{g}\left(0,t\right)=\frac{\alpha_{g,7}\left(t\right)}{\alpha_{g,6}}$$

(A9) $$T_{g}\left(L,t\right)=\frac{1}{1-0.5L_{z}\alpha_{g,5}}\left[0.5L\;T_{g,\infty }\left(\alpha_{g,4}+\alpha_{g,5}\right)-\left(1+0.5L\;\alpha_{g,4}\right)\frac{\alpha_{g,7}\left(t\right)}{\alpha_{g,6}}\right]$$

### Appendix A.3. CIEA Method’s Application to the Energy Balance of the Solid Phase in Reaction Zone from PBMR

The boundary conditions (Equations (33) and (34)) of the energy balance for the endothermic reactions at the inlet and outlet of the reaction zone from PBMR were used to transform Equation (30) into Equation (48). The coefficients from Equation (48) as well as expressions of T_er_ (0, t) and T_er_ (L, t) are reported as follows.

(A10) $$\beta_{\mathrm{er},1}=\frac{\lambda_{s,\mathrm{eff}.}}{\rho_{s}C_{p,s}};\;\beta_{\mathrm{er},2}=h_{\mathrm{sg}}\frac{6}{d_{p}}\frac{1}{\rho_{s}C_{p,s}}\frac{\left(1-\varepsilon_{b}\right)}{\varepsilon_{b}};\;\beta_{\mathrm{er},3}=\frac{\left(1-\varepsilon_{p}\right)}{\varepsilon_{p}}$$

(A11) $$\beta_{\mathrm{er},4}=\frac{q_{h}}{\lambda_{s}};\;\beta_{\mathrm{er},5}=\frac{h_{\mathrm{sg},\mathrm{eff}.}}{\lambda_{s,\mathrm{eff}.}};\;\beta_{\mathrm{er},6}=2L\left[3-\frac{\left(6+L\beta_{s,5}\right)}{2+L\beta_{s,5}}\right]$$

(A12) $$\beta_{\mathrm{er},7}\left(t\right)=12{\bar{T}}_{\mathrm{er}}\left(t\right)-L^{2}\left(\beta_{\mathrm{er},4}-\beta_{\mathrm{er},5}T_{\mathrm{er},\infty }^{\mathrm{Bot}.}\right)-\frac{L^{2}\left(6+L_{z}\beta_{\mathrm{er},5}\right)\left(\beta_{\mathrm{er},4}+\beta_{\mathrm{er},5}T_{\mathrm{er},\infty }^{\mathrm{Bot}.}\right)}{2+L\;\beta_{\mathrm{er},5}}$$

(A13) $$T_{\mathrm{er}}\left(0,t\right)=\frac{\beta_{\mathrm{er},7}\left(t\right)}{\beta_{s,6}};\;T_{\mathrm{er}}\left(L,t\right)=\frac{1}{2+L\beta_{\mathrm{er},5}}\left[L_{z}\left(\beta_{\mathrm{er},4}+\beta_{\mathrm{er},5}T_{\mathrm{er},\infty }^{\mathrm{Bot}.}\right)+2\frac{\beta_{\mathrm{er},7}\left(t\right)}{\beta_{\mathrm{er},6}}\right]$$

### Appendix A.4. CIEA Method’s Application to the Transport of Components i in Reaction Zone from PBMR

The boundary conditions (Equations (37) and (38)) of the mass balance for components i (i = CH_4_, H_2_O, CO and CO_2_) at the inlet and outlet of the reaction zone from PBMR have been used to convert Equation (35) into Equation (49). The coefficients from Equation (49) as well as expressions of F_i_ (0, t) and F_i_ (L, t) are described as follows.

(A14) $$\varphi_{f,1}=\frac{4q_{g}}{S_{\mathrm{sp}}\pi d_{\mathrm{rz}}^{2}}\frac{g}{u_{\mathrm{sg}}};\;\varphi_{f,2}=\frac{g}{u_{\mathrm{sg}}}\frac{D_{\mathrm{ax}.,i}}{S_{\mathrm{sp}}};\;\varphi_{f,3}=\frac{g}{u_{\mathrm{sg}}}\rho_{s}r_{\mathrm{rz}}^{2}L\left(1-\varepsilon_{b}\right);\;\varphi_{f,4}=\frac{L}{\varepsilon_{b}}\frac{u_{\mathrm{sg}}}{D_{\mathrm{ax}.,i}}$$

(A15) $$\varphi_{f,5}=\frac{L}{\varepsilon_{b}}\frac{k_{\mathrm{sg}}}{D_{\mathrm{ax}.,i}};\;\varphi_{f,6}=L\left(6+L\varphi_{f,4}\right)-\frac{L\left(6-L\varphi_{f,5}\right)}{2-L\varphi_{f,5}}\left(2+L\varphi_{f,4}\right)$$

(A16) $$\varphi_{f,7}\left(t\right)=12{\bar{F}}_{i}\left(t\right)-L^{2}\left(\varphi_{f,5}F_{i,\infty }^{\mathrm{Up}.}-\varphi_{f,4}F_{i,\infty }^{\mathrm{Bot}.}\right)-\frac{L^{2}\left(6-L\varphi_{f,5}\right)\left(\varphi_{f,4}F_{i,\infty }^{\mathrm{Up}.}+\varphi_{f,5}F_{i,\infty }^{\mathrm{Bot}.}\right)}{2-L\varphi_{f,5}}$$

(A17) $$F_{i}\left(0,t\right)=\frac{\varphi_{f,7}\left(t\right)}{\varphi_{f,6}}$$

(A18) $$F_{i}\left(L,t\right)=\frac{1}{2-L\varphi_{f,5}}\left[\left(2+L\varphi_{f,4}\right)\frac{\varphi_{f,7}\left(t\right)}{\varphi_{f,6}}-L\left(\varphi_{f,4}F_{i,\infty }^{\mathrm{Up}.}+\varphi_{f,5}F_{i,\infty }^{\mathrm{Bot}.}\right)\right]$$

### Appendix A.5. CIEA Method’s Application to the Transport of H_2_ in Reaction Zone from PBMR

The boundary conditions (Equations (41) and (42)) of the mass balance of H_2_ at the inlet and outlet of the reaction zone from PBMR were used to convert Equation (39) into Equation (50). The coefficients from Equation (50) as well as expressions of $F_{H_{2}}\left(0,t\right)$ and $F_{H_{2}}\left(L,t\right)$ are depicted as follows.

(A19) $$\vartheta_{H_{2},1}=\frac{g}{u_{\mathrm{sg}}}\frac{4q_{g}}{S_{\mathrm{sp}}\pi d_{\mathrm{rz}}^{2}};\;\vartheta_{H_{2},2}=\frac{g}{u_{\mathrm{sg}}}\frac{D_{\mathrm{ax},H_{2}}}{S_{\mathrm{sp}}};\;\vartheta_{H_{2},3}=\frac{g}{u_{\mathrm{sg}}}\rho_{s}r_{\mathrm{rs}}^{2}L\left(1-\varepsilon_{b}\right)$$

(A20) $$\vartheta_{H_{2},4}=\frac{g}{u_{\mathrm{sg}}}\pi d_{\mathrm{rs}}^{2}J_{H_{2},\mathrm{per}.};\;\vartheta_{H_{2},5}=\frac{L}{\varepsilon_{b}}\frac{u_{\mathrm{sg}}}{D_{\mathrm{ax},H_{2}}};\;\vartheta_{H_{2},6}=\frac{L}{\varepsilon_{b}}\frac{k_{\mathrm{sg},\mathrm{eff}.}}{D_{\mathrm{ax},H_{2}}}$$

(A21) $$\vartheta_{H_{2},7}=L\left(6+L\;\vartheta_{H_{2},5}\right)-\frac{L\left(6-L\;\vartheta_{H_{2},6}\right)}{2-L\;\vartheta_{H_{2},6}}\left(2+L\;\vartheta_{H_{2},5}\right)$$

(A22) $$\begin{array}{ll} \vartheta_{H_{2},8}\left(t\right)= & 12{\bar{F}}_{H_{2}}\left(t\right)-L^{2}\left(\vartheta_{H_{2},6}F_{H_{2},\infty }^{\mathrm{Bot}.}-\vartheta_{H_{2},5}F_{H_{2},\infty }^{\mathrm{Up}.}\right)-\frac{L\left(6-L\vartheta_{H_{2},6}\right)}{2-L\vartheta_{H_{2},6}}L\\ & \left(\vartheta_{H_{2},5}F_{H_{2},\infty }^{\mathrm{Up}.}+\vartheta_{H_{2},6}F_{H_{2},\infty }^{\mathrm{Bot}.}\right) \end{array}$$

(A23) $$F_{H_{2}}\left(0,t\right)=\frac{\vartheta_{H_{2},8}\left(t\right)}{\vartheta_{H_{2},7}}$$

(A24) $$F_{H_{2}}\left(L,t\right)=\frac{1}{2-L\vartheta_{H_{2},6}}\left[\frac{\left(2+L\vartheta_{H_{2},5}\right)\vartheta_{H_{2},8}\left(t\right)}{\vartheta_{H_{2},7}}-L\left(\vartheta_{H_{2},5}F_{H_{2},\infty }^{\mathrm{Up}.}+\vartheta_{H_{2},6}F_{H_{2},\infty }^{\mathrm{Bot}.}\right)\right]$$
